# Exopolysaccharide from marine microalgae belonging to the *Glossomastix* genus: fragile gel behavior and suspension stability

**DOI:** 10.1080/21655979.2023.2296257

**Published:** 2023-12-28

**Authors:** Virginie Dulong, Christophe Rihouey, Clément Gaignard, Nicolas Bridiau, Priscilla Gourvil, Céline Laroche, Guillaume Pierre, Tony Varacavoudin, Ian Probert, Thierry Maugard, Philippe Michaud, Luc Picton, Didier Le Cerf

**Affiliations:** a Université de Rouen Normandie, INSA Rouen Normandie, CNRS, PBS Laboratory, Rouen, France; b Université Clermont Auvergne, Clermont Auvergne INP, CNRS, Institut Pascal, Clermont-Ferrand, France; c La Rochelle Université, CNRS, LIENSs Laboratory, La Rochelle, France; d Station Biologique de Roscoff (SBR), Sorbonne Université, CNRS, Roscoff, France

**Keywords:** Polysaccharide, microalgae, *Glossomastix*, fragile gel, stabilizer

## Abstract

With the aim to find new polysaccharides of rheological interest with innovated properties, rhamnofucans produced as exopolysaccharides (EPS) in a photobioreactor (PBR) and an airlift bioreactor (ABR) by the marine microalgae Glossomastix sp. RCC3707 and RCC3688 were fully studied. Chemical characterizations have been conducted (UHPLC – MS HR). Analyses by size-exclusion chromatography (SEC) coupled online with a multiangle light scattering detector (MALS) and a differential refractive index detector showed the presence of large structures with molar masses higher than 10^6^ g.mol^−1^. The rheological studies of these EPS solutions, conducted at different concentrations and salinities, have evidenced interesting and rare behavior characteristic of weak and fragile hydrogels i.e. gel behavior with very low elastic moduli (between 10^−2^ and 10 Pa) and yield stresses (between 10^−2^ and 2 Pa) according to the EPS source, concentration, and salinity. These results were confirmed by diffusing wave spectroscopy. Finally, as one of potential application, solutions of EPS from Glossomastix sp. have evidenced very good properties as anti-settling stabilizers, using microcrystalline cellulose particles as model, studied by multiple light scattering (MLS) with utilization in cosmetic or food industry. Compared to alginate solution with same viscosity for which sedimentation is observed over few hours, microalgae EPS leads to a stable suspension over few days.

## Graphical Abstract

**Figure:**
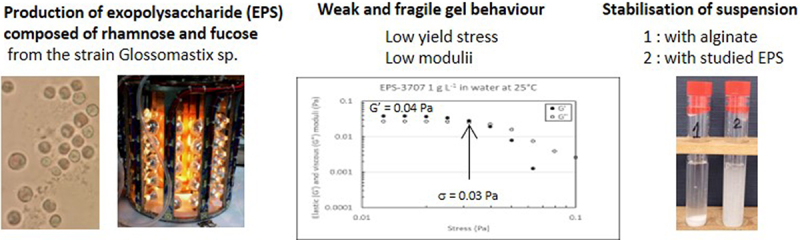

## 1. Introduction

Microalgae are unicellular photosynthetic microorganisms that produce valuable and diverse compounds such as lipids, proteins, pigments, and polysaccharides [1,2], and can also present new sustainable food and feed sources in circular bioeconomy [3,4]. These abundant biological compounds have received great attention since the detection of their different biological activities [5]. Microalgal polysaccharides have few commercial applications, even though their production, chemical characterization and biological activities are now widely reported in the literature [6]. This can be easily explained by the costs of microalgae production, which are linked to photoproduction, harvesting in diluted media and the difficulty of refining the biomass [7]. For the extraction of EPSs after production, several methods are generally used, such as dialysis, alcohol precipitation and tangential ultrafiltration. A compromise has then to be done between the purity and the cost [8]. The physiological functions of these polysaccharides are extremely diverse and depend on their sugar composition, their structure and the presence of specific chemical functions, such as those carried out by sulfate and acetyl groups [5]. Some microalgae produced intracellular polysaccharides, such as starch, floridean starch, paramylon or chrysolaminarin, are clearly carbon and energy reserves. Others are well known as cell wall components (β-(1,4/1,3) glucans, for example). However, the physiological functions of extracellular polysaccharides remain unclear. These polysaccharides are associated with cell walls and/or excreted in the surrounding environment (exopolysaccharides (EPS)), depending both on normal physiological processes and under conditions of physiological stress [9]. Among the microalgae studied for the production of exopolysaccharides, data are available in the literature for *Dinoflagellates, Prymnesiophyta, Prasnophyta, Miozoa, Haptophyta, Chlorophyta, Charophyta, Bacillariophyta,* and above all *Rhodophyta* [10–12]. Among Rhodophyta, microalgae belonging to the *Porphyridium* [13,14] or *Rhodella* [15,16] genera have been abundantly described as EPS producers, and some of these microalgae have been exploited in the cosmetic industry for their photoautotrophic production. These exopolysaccharides are mainly sulfate galactoxylans with rheological properties [14] comparable to the polysaccharides used industrially as alginates, for example.

Indeed, alginate but also xanthan, guar, pectin, etc., are hydrocolloids widely used by the cosmetics or food industry as additives for texture formulation [17], with the solutions behaving as shear thinning pseudoplastic fluids with a sensitivity to temperature. By adjusting the polysaccharide concentration and depending on the molecular weights and chain conformations, it is possible to obtain various textures, such as those produced by viscous, viscoelastic, and weak gel behaviors [18,19]. Additionally, entangled polymer chains slow the dynamics of particles in suspension and delay the phenomena of sedimentation and creaming [20]. Nevertheless, the best rheological behavior leading to the best anti-settling properties together with shear thinning characteristics under low stress are difficult to obtain. Some studies report such atypical behavior, sometime called ‘fragile gels’, but they are mainly based of suitable and specific polysaccharide mixtures [21].

In a previous study, Gaignard et al. [22] screened 166 marine microalgae and cyanobacteria from the Roscoff Culture Collection (France) for the production of EPS. Forty-five strains were identified as EPS producers, highlighting 8 new genera of microalgae with the ‘EPS+’ phenotype. All the EPSs characterized appeared as heteropolysaccharides with complex structures, including up to 6 different monosaccharide species. Certain EPSs inhibited human matrix metalloproteinase-1 and their depolymerized forms highly stimulated collagen production by human dermal fibroblasts [23]. In light of the above considerations, EPSs produced by two newly identified strains of *Glossomastix* have been selected to study in depth their behavior in solution. They were mainly composed of fucose (40–56%) and rhamnose (20–31%) and other minor monosaccharides, such as uronic acids [22]. Despite these two strains of microalgae belong to the same genus and produced EPSs with very similar compositions, they were not equally sensitive to mechanical agitation. Indeed, the strain RCC 3707 could only grow in airlift reactors whereas biomass from the strain RCC 3688 was classically obtained in stirred photobioreactor. Consequently, the behavior in water solution of the two EPSs was studied using rheology and diffusing wave spectroscopy to identify some differences between them explaining putative physiological functions as strain protector against shear stress.

The rheological investigation studied as a function of polysaccharide concentration, different lyotropic or chaotropic salts and ionic strength have evidences atypical properties of very weak gel consistent with fragile structure. Thus, their capacity to stabilize suspensions was explored with a model system composed of microcrystalline cellulose particles.

## 2. Materials and methods

### 2.1. Origin of strains

*Glossomastix sp*., RCC3707, and RCC3688, which are classified in the *Pinguiophyceae*, were from the Roscoff Culture Collection (RCC) France [24].

### 2.2. Production of EPS

The strain *Glossomastix* sp. RCC3707 was cultivated under airlift conditions in bottles (5 L) to overcome its sensitivity to mechanical agitation. Indeed, contrary to the strain RCC3688, the strain RCC3707 did not grow under stirring conditions due to shear stress. The airlift cultures were equipped with a cylindrical air diffuser connected to a compressed air network sterilized by filtration (0.22 µm). The cultures were performed at 20°C (air-conditioned room) and illuminated by neon lights providing an irradiance of 40 µmol photons.m^−2^ s^−1^. The strain was grown in batch mode and under photoautotrophic conditions with a 16 h/8 h day/night cycle. The 5 L batch cultures were grown in ‘f/2 enriched’ medium for 50 days after inoculation with 500 mL of exponential phase culture [20].

The strain RCC3688, which is less sensitive to mechanical agitation, was cultivated for 50 days in a 5 L cylindrical photobioreactor (radius 0.08 m) containing 4.2 L of ‘f/2 enriched’ medium and inoculated with 800 mL of exponential phase culture [20]. Continuous artificial illumination was provided by 55 halogen lamps (Sylvania Professional 25, BAB 38°, 12 V, 20 W) surrounding the culture and allowing control of the irradiance between 150 and 300 µmol photons m^−2^ s^−1^. The agitation speed was fixed at 120 rpm, and the temperature was 20°C depending on the experiment. Photosynthetic activity was monitored using a pO_2_ sensor, and the pH was maintained at 8 by varying the CO_2_ content (between 0.5 and 3%) in continuously injected sterile air (100 mL min^−1^).

Growth kinetics of cultures into airlift reactor and cylindrical photobioreactor were not possible using classical measurements (turbidimetry, Mallasez cells or dried weight quantification of biomass) because of cell aggregates occurring during culture.

### 2.3. Extraction of EPS

Cultures supplemented with 2 volumes of sterile culture medium were centrifuged at 14,000 × g for 20 min at 25°C. The EPS in the supernatants were desalted by cycles of concentration dilution (Milli-Q water) using a Vivaflow 200 filter equipped with a polyethersulfone 50 kDa NMWCO membrane (Sartorius, Germany). The EPSs were considered purified when the conductivity of the filtrates was near that of Milli-Q water (approximately 2 µS cm^−1^). Finally, the EPS solutions were freeze-dried for 48 h, and the samples were stored at room temperature until further analyses. After extraction, the EPS yields were 0.65 and 1.5 g L^−1^ for strains RCC3707 and RCC3688, respectively.

### 2.4. Biochemical composition

The total carbohydrate concentration was determined by the colorimetric method of Dubois using the phenol and sulfuric acid assay [25]. Total neutral sugar and uronic acid contents were quantified with a UV-Vis spectrophotometer using the sulfuric resorcinol method [26] and the *m*-hydroxydiphenyl (MHDP) assay [27], respectively. The results were expressed in equivalents of d-glucose for the neutral sugars, while d-glucuronic acid was used as a standard for uronic acids using the correction factors of sugar concentrations [28]. Protein concentrations were determined according to the Bradford method (Coomassie Brilliant Blue G-250 method) using Bio-Rad reagent and bovine serum albumin (BSA) as a standard [29]. The sulfation degree of the polysaccharides was evaluated according to the turbidimetric method (BaCl_2_/gelatin) [30].

### 2.5. Depolymerization procedure by high-pressure pre-treatment followed by solid acid-catalysed hydrolysis

The first step of the depolymerization method consisted of a high-pressure pre-treatment aimed at reducing EPS viscosity in aqueous solution and initiating their depolymerization [23,31]. This step was carried out using a TS Haiva series 2.2-kW high-pressure grinder (Constant Systems Ltd., UK), usually designed for cell lysis. First, EPS solution at 20 mg mL^−1^ was placed in the grinder, treated at the maximal pressure of 2.7 kbar during one run and transferred to a storage tank. Then, 10 mL of deionized water was injected into the grinder to wash it and thus counteract the loss of EPS material that was probably occurring during the treatment. These two steps were repeated four times, and then the washing waters and EPS solutions were collected and freeze-dried at −80°C using a Heto PowerDry OL 6000 lyophilization system (Thermo Electron Co., France).

Secondly, 25 ml of high pressure-pre-treated EPS solution at 2 mg mL^−1^ was placed in a 50 mL bottle containing 10 g of a strong acidic cation-exchange resin (Amberlyst^TM^ 15 dry; 4.7 meq H+/g _dry material_), previously hydrated and washed with ultrapure water [23]. This batch system was then hermetically sealed and placed in an LSE cabinet-style shaking incubator (Corning, NY, USA) and maintained at 80°C under orbital shaking at 120 rpm. After 48 h, the medium was frozen in an ice bath and centrifuged at 10,000 × g for 5 min at room temperature. The supernatant was then neutralized at pH 7.0 by adding 1 M NaOH and was freeze-dried at −80°C using a Heto PowerDry OL 6000 lyophilization system (Thermo Electron Co., France) prior to UHPLC-HRMS HR analysis.

### 2.6. Structural characterization by UHPLC-HRMS (ultrahigh-pressure liquid chromatography-high resolution mass spectrometry)

Analyses were carried out using an ‘Acquity UPLC H-class’ UHPLC system (Waters, CT, USA) coupled to a ‘XEVO G2-S QTof’ high-resolution mass spectrometer equipped with an electrospray ionization (ESI) source (Waters, UK). The UHPLC system consisted of a quaternary pump (Quaternary Solvent Manager, Waters) and an autosampler (Sample Manager-FTN, Waters) equipped with a 10 µL injection loop. After dissolution in an aqueous solution of 5 nM heptyl ammonium formate at 1 mg mL^−1^, 5 µL of hydrolyzed EPS was injected into an Acquity UPLC BEH C18 column (50 mm x 2.1 mm, 1.7 µm; Waters) heated at 45°C. The system was operated at 0.3 mL min^−1^ under the following gradient elution program involving solvent A (5 nM heptyl ammonium in H_2_O, 0.0238% (v/v) formic acid, pH 4.0) and solvent B (5 nM heptyl ammonium in MeOH/H_2_O (3:1, v/v), 0.0238% (v/v) formic acid, pH 4.0) as follows: 0–2 min, 10% B; 2–4 min, 10–25% B; 4–8 min, 25% B; 8–10 min, 25–50% B; 10–14 min, 50% B; 14–15 min, 50–100% B; 15–19 min, 100% B; 19–20 min, 100–10% B; 20–25 min, 10% B. The column and the autosampler were maintained at + 45°C and + 8°C, respectively. Eluted compounds were ionized in the negative mode (ESI-) with the following parameters: capillary voltage 2.5 kV, source temperature 120°C, source offset 80 V, desolvation temperature 500°C, cone gas flow 50 L h^−1^, and desolvation gas flow 1000 L h^−1^.

The MS and MS/MS experiments were performed using either the MS^E^ (Waters, UK) or targeted MS/MS approaches in centroid mode with a scan time of 0.5 s [32]. The MS^E^ experiment consists of data acquisitions in a single same run, with no collision energy in function 1 so that no fragmentation occurs (low energy function) and a ramped collision energy in function 2 to generate fragment ions (high energy function). MS^E^ software algorithms then assign fragment ion spectra to their associated ion precursor peak by profiling each chromatographic peak and determine their retention time. A targeted MS/MS experiment consists of acquisition in a single run, with selection of the targeted precursor ion and a specific collision energy to generate fragment ions. MS^E^ function 2 acquisitions were performed with a ramped collision energy of 15–60 eV. Targeted MS/MS acquisitions were performed with various collision energies depending on the targeted ion, as specified in the text.

Leucine enkephalin (*M* = 555.62 Da, 1 ng µL^−1^) was used as a lock-mass for mass shift correction, and the mass spectrometer was calibrated before analyses using 0.5 mM sodium formate solution.

### 2.7. Macromolecular characterization

The EPS absolute average number and weight molar masses (M_n_ and M_w_, respectively) and molar mass distribution were determined using online coupling of a size exclusion chromatograph (SEC), a multiangle light scattering system (MALS) (Dawn HELEOS II, Wyatt Technology, Inc., USA), a viscosity detector (VD) (ViscoStar II, Wyatt Technology, Inc., USA) and a differential refractive index detector (DRI) (RID-10A Shimadzu, Japan). The MALS was fitted with a K5 cell of 50 μL and 18 photodiodes (normalized relative to the 90° detector using bovine serum albumin). The SEC line consists of a degasser (DGU-20A3 Shimadzu, Japan), a pump (LC10Ai Shimadzu, Japan) at a 0.5 mL min^−1^ flow rate, an OHPAK SB-G guard column for protection and two OHPAK SB 804 and 806 HQ columns (Shodex Showa Denko K.K., Japan) in series packed with a poly(2-hydroxyethyl methacrylate) gel. 0.1 M LiNO_3_, used as the eluent, was previously filtered through a 0.1 μm filter unit (Millipore, USA). Samples for analysis were prepared from lyophilized products (0.3 g L^−1^ in LiNO_3_ 0.1 M) with stirring at 500 rpm for 72 h at room temperature. Five hundred microliters of a 0.45 μm filtrated sample solution (Millipore, USA) was injected with an automatic injector (SIL-20A Shimadzu, Japan). All analyses were treated using the Astra 6.1.7.16® software package via a data processing Zimm order 1 with angles between 37.5° and 80.1° [33]. The concentration of each eluted fraction was determined with the DRI detector with an increment refractive index dn/dC 0.15 mL g^−1^ to obtain the number and weight average molar masses (M_n_ and M_w_) and the average intrinsic viscosity [ŋ] [34].

### 2.8. Rheology

Rheological measurements were performed with a DHR2 rheometer (TA Instrument, U.K.), using a standard-size double concentric cylinder geometry (aluminum, gap 500 μm, internal diameter 32 mm external diameter 35.02 mm) or a cone-plate geometry (diameter 40 mm; angle 2°; gap 57 μm) for higher concentrations. The choice of the geometry depends on the viscosity of the solution and thus, in our case, on the EPS concentration. All measurements were performed with a solvent trap to prevent any evaporation.

The EPS solutions were prepared by adding lyophilized EPS in pure water or in water with salt at the desired concentration under vigorous stirring for 72 h at room temperature.

Flow procedures were performed at 25°C from 0.1 to 1000 s^−1^ (during 10 min) and from 1000 to 0.1 s^−1^ (during 10 min) at various concentrations in water or in salt media (NaCl or KSCN). Oscillation procedures were performed at 25°C, 37°C, or 60°C. The linearity domain of each EPS solution was determined before performing the frequency sweeps (between 0.001 or 0.01 and 10 Hz). The limit value of stress (σ_lim_) of the linearity domain was graphically determined. The power law model (Equation 1) and the Herschel-Bulkley model (Equation 2) were applied to the flow curves to further explore the behavior under stress [35].

1 $$\sigma =K{\dot{\gamma }}^{n}$$

2 $$\sigma =\sigma_{0}+K{\dot{\gamma }}^{n}$$

where σ is the shear stress, $\dot{\gamma }$ is the shear rate, $\sigma_{0}$ is the yield stress, K is the consistency index, and n is the flow behavior index.

The analyses were performed with TRIOS V 3.1.0.3538 rheology software (TA Instrument, U.K.).

### 2.9. Diffusing Wave Spectroscopy (DWS)

The viscoelastic properties of EPS solutions were also evaluated in a nondestructive manner (without any mechanical stress) using diffusing wave spectroscopy (DWS) (Rheolaser Master™, Formulaction, France), thus measuring the particles’ Brownian motion, which depends on the viscoelastic feature of the sample [36,37]. Microrheological measurements were taken by adding latex beads (1 μm, 0.1 wt. %).

The mean square displacement (MSD) was obtained from the microrheological analysis. The MSD was used to determine the sample particle movement as a function of time. The microrheological parameters, namely, MSD curves, elasticity index (EI) and solid liquid balance (SLB), were collected and processed by RheoSoft Master 1.4.0.0. The EI value is the sample elasticity strength and corresponds to the MSD slope at a short decorrelation time. SLB is the ratio between the solid-like and liquid-like behavior of the sample. Namely, 0.5 < SLB < 1 indicates that the liquid behavior dominates, while 0 < SLB < 0.5 indicates that the solid behavior dominates, thus defining it as gel behavior [38,39].

EPS was dissolved in 20 mL of water at 0.1, 0.5, 0.75 or 1 g L^−1^. A commercial alginate (Cargill, France) was used at 1 g L^−1^ for comparison as a conventional viscosifier. Then, 200 µL of latex particle solution (10% v/v, latex particle size of 1 µm) was added to the polysaccharide solution, and the Brownian motion of the latex particles was studied. Measurements were performed three times at 25, 37 or 60°C for 2 h. The results (EI and SLB) are given as the mean of three measurements.

### 2.10. Multiple Light Scattering (MLS)

Stabilisation of suspension was studied using multiple light scattering (MLS) (Turbiscan™ Classic MA2000, Formulaction (France)). This technique is based on the variation in backscattered or transmitted light signals. The transmitted (T) intensities are measured versus sample height and time. For analyses, the percentage of transmission at the bottom (2 cm) and at the top of the tube (5.5 cm) was determined as a function of time. If the percentage of transmission increases and reaches a progressive value close to 100% in all the tubes, sedimentation occurs. In the opposite case, it is creaming that occurs [40].

Suspensions of microcrystalline cellulose (ACROS, France, average particle size 90 µm) were prepared by mixing 0.1 g of microcrystalline cellulose in 6 mL of polysaccharide solutions at different concentrations in water.

## 3. Results and discussion

### 3.1. Biochemical composition

The biochemical compositions of the two isolated EPSs were investigated using colorimetric assays, and their purities were measured depending on their monosaccharide compositions determined using GC‒MS analysis (Table 1).

**Table 1.** Biochemical composition of EPS from Glossomastix sp. RCC3707 and RCC3688.

EPS	Purity (%)	Total sugars (%)	Neutral sugars (%)*	Uronic acids (%)*	Proteins (%)	Sulfates (%)
3688	86	69.9	81.0	18.9	7.7	8.9
3707	61	48.4	53.0	47.0	4.4	13.7

*As part of total sugar.

The two EPSs with purities between 61 and 86% depending on their monosaccharide contents were mainly composed of carbohydrates (48.4–69.9%), principally neutral (81–53%), sulfates (13.7–8.9%) and minor amounts of proteins (4.4–7.7%).

An UHPLC‒MS HR analysis was first performed on hydrolyzed EPS-3707 using an ion-coupled reversed-phase C18 column (heptyl ammonium formate- RPIR) coupled to an ESI-qTOF MS in negative polarity. Due to the complexity of the oligosaccharide structures and the multiple possibilities (Table S1), special attention was given to 7 specific ions that were selectively analyzed by targeted MS/MS (Table 2, Figure S1, Table S2). The results confirmed the previously published monosaccharide composition with rhamnose and fucose as major monosaccharides and galactose, galacturonic and glucuronic acids as minor monosaccharides [22]. In addition, the results also highlight the diversity of the monosaccharide chains, with repeated units of Rha/Fuc-GalA/GlcA or Rha/Fuc or GalA/GlcA with variable lengths, leading us to conclude that the chemical structure of these EPSs is complex.

**Table 2.** Oligosaccharide sequences identified by ultra-high-pressure liquid chromatography-mass spectrometry untargeted in-source fragmentation (UHPLC-MS2) from EPS-3707.

	Ion (m/z)					Candidate		
t_R_	*Experimental value*	*Predicted value*	Resolution (ppm)	Ionized adduct	Adduct molecular formula	*Sequence* ^*a*^*right read direction* ^*b*^*two possible read directions*	*DP*	Sulfate groups
0.983	679.1935	679.1932	0.44	[2M-H]^–^	[C_24_H_39_O_22_]^−^	Rha/Fuc-GalA/GlcA^a^	2	0
1.52	535.1371	535.1333	7.10	[M-H]^−^	[C_18_H_31_O_16_S]^−^	Rha/Fuc-Rha/Fuc-Rha/Fuc-(OSO_3_^−^)^b^	3	1
2.11	973.309	973.307	2.05	[M-H]^−^	[C_36_H_61_O_28_S]^−^	Rha/Fuc-Rha/Fuc-Rha/Fuc-Rha/Fuc-Rha/Fuc-Rha/Fuc(+ OSO_3_^−^)^a^	6	1
5.6	1149.3435	1149.3391	3.83	[M-H]^−^	[C_42_H_69_O_34_S]-	GalA/GlcA-Rha/Fuc-Rha/Fuc-Rha/Fuc-Rha/Fuc-Rha/Fuc-Rha/Fuc(OSO_3_^−^)^a^	7	1
12.11	1085.2172	1085.211	5.71	[M+Na-2H]^−^	[C_36_H_54_O_34_NaS]^−^	GalA/GlcA-Rha/Fuc-GalA/GlcA-Rha/Fuc-GalA/GlcA-Rha/Fuc (+ OSO_3_^−^Na^+^)^b^	6	1
12.31	773.1838	773.1823	1.94	[M-2H]^2-^	[C_52_H_82_O_49_S]^2-^	GalA/GlcA-Rha/Fuc-Gal/Glc-GalA/GlcA-Rha/Fuc-Rha/Fuc(OSO_3_^−^)-GalA/GlcA-Rha/Fuc-GalA/GlcA^b^	9	1
16.57	1137.2118	1137.2189	6.24	[M-2H]^2-^	[C_78_H_108_O_75_]^2-^	(GalA/GlcA)_2_-Rha/Fuc-(GalA/GlcA)_10_^b^	13	0

Rha : Rhamnose; Fuc : Fucose; GalA : galacturonic acid ; GlcA : Glucuronic acid; Gal : Galactose; Glu : glucose.

*rhamnose/fucose, galacturonic acid/glucuronic acid and galactose/glucose being isobaric, they cannot be distinguished by MS.

### 3.2. Macromolecular characteristics

**Figure 1.**
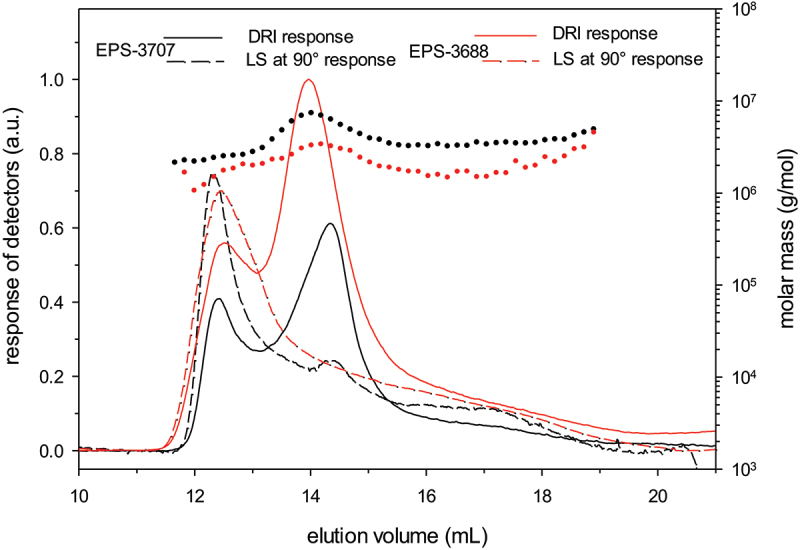
Elution profiles of EPSs (LiNO_3_ at 0.1 mol L^−1^): Light Scattering signal (LS), dash line curves; Differential Refractive Index (DRI), full line curves; and molar mass distributions.

The main difficulties encountered during these types of analyses are high filtration losses, separation and detection problems. Several EPS concentrations, injected volumes, eluents (nature of the salt, ionic strength and pH) and flow rates were tested to limit these problems. The best results were obtained for the two EPSs with a concentration of 0.3 g L^−1^ and an injection volume of 500 μL with an eluent composed of 0.1 mol L^−1^ LiNO_3_ without controlled pH (Figure 1). The recovery rate at the exit of the columns was 50%, but the separation was not sufficient. For both analyzed EPSs, the molar masses are higher than 10^6^ g mol^−1^, and the intrinsic viscosities are higher than 1 000 mL g^−1^ with hydrodynamic radii higher than 90 nm. According to the recovery rate after 0.45 µm filtration (i.e. ~50%), one can consider that a large part of the samples exhibited a very large structure that could consist of large aggregated polysaccharides associated via possible hydrophobic interactions.

The difficulties in characterizing EPSs produced by microalgae such as *Porphyridium* sp. have already been reported in the literature. Guereh et al. [41] have shown that ultrasound treatment could breakdown the chains, but this treatment had to be very short; otherwise, it would be accompanied by degradation of the chains with breakage of their glycosidic bonds. Gargouch et al. [42] subjected their EPS solutions to high-pressure treatment. The aggregates did disappear, but the chains were severely degraded.

### 3.3. Rheological properties

The rheological properties of the EPS solutions in water or in saline medium were studied by rheology in flow experiments leading to the viscosity properties or in oscillation experiments to analyze the viscoelastic properties.

**Figure 2.**
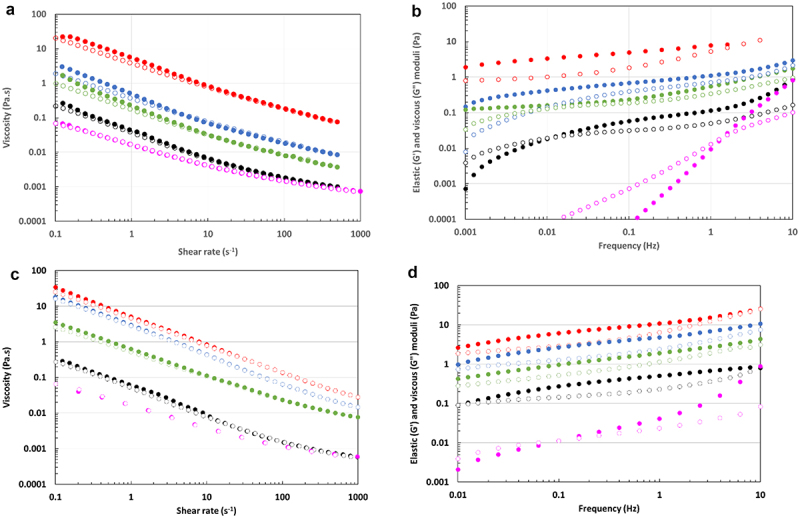
a) Viscosity of EPS-3707 (upward: fill circles; downward: blank circles) b) elastic and viscous moduli of EPS-3707 (G’ fill and G’’ blank circles) c) viscosity of EPS-3688 (upward: fill circles; downward: blank circles) d) elastic and viscous moduli of EPS-3688 (G’ fill and G’’ blank circles) colour for the symbols : 20 g L^−1^ (red), 10 g L^−1^ (blue), 5 g L^−1^ (green), 1 g L^−1^ (black) and 0.5 g L^−1^ (pink) in water at 25°C.

The rheological behaviors of EPS-3688 and EPS-3707 solutions (Figure 2) were also studied at different concentrations. They displayed non-Newtonian behavior without real thixotropy and with no Newtonian plateau at low shear rates, consistent with a yield stress fluid. Figure 2(a,c).

The power law and the Herschel-Bulkley model were applied to these flow curves to determine whether the EPS solutions exhibited a yield stress σ_0_. The obtained R^2^ values, summarized in Table S3, were always up to 0.99 with the Herschel-Bulkley model, showing a yield stress behaviour. For the power-law model, the R^2^ was slightly smaller (from 0.95 to 0.91) due to very low values of yield stress (Table 3).

**Table 3.** Comparison between the yield stress (σ_0_) and the limit stress (σ_lim_) and characteristics at the cross point for the two EPSs at different concentrations in water at 25°C.

	Concentration (g L^−1^)	0.5	1	5	10
EPS-3707	^σ^_0_^a^ (Pa)	0.010 ± 0.001	0.030 ± 0.002	0.18 ± 0.02	0.37 ± 0.04
	σ_lim_ (Pa)	0.006 ± 0.001	0.03	0.2 ± 0.001	0.4 ± 0.001
EPS-3688	σ_0_^a^ (Pa)	0.010 ± 0.002	0.050	0.30 ± 0.04	1.0± 0.1
	σ_lim_ (Pa)	0.020 ± 0.002	0.040	1.3 ± 0.1	2.3 ± 0.2
EPS-3707	t_r_ (s)	0.8 ± 0.2	85	140 ± 30	>1000
	G’ (Pa)	0.010 ± 0.001	0.02	0.12 ± 0.01	–
EPS-3688	t_r_ (s)	10 ± 1	100	>100	>100
	G’ (Pa)	0.010 ± 0.001	0.090	–	–

determined according to Herschel-Bulkley model (Equation 2).

The frequency sweeps of the EPS-3707 Figure 2(b) and EPS-3688 Figure 2(d) solutions showed atypical behaviour. Indeed, at 20, 10, 5 and 1 g L^−1^, an elastic to visco-elastic behaviour was observed between 0.01 and 10 Hz, with low moduli but G’ higher than G’’ on the whole frequency sweep for the higher concentrations. The tangent of the phase angle (δ) between stress and strain (tan δ = G’‘/G’) was never lower than 0.1, showing weak gel-like behavior. A good correlation was obtained between the yield stress and the limit stress of the linearity domain of the oscillation procedure. These characteristics indicated the behavior of a weak fragile gel with a low value for the limit stress.

For viscoelastic mechanical spectra, the relaxation time t_r_, or disentanglement time (defined as the inverse number of the frequency where G’ is equal to G’’ and given in Table 3), and curves in Figure 2(b,d) confirmed the dynamics of this weak gel.

Regarding the studied EPS concentration range, the observed rheological behaviors (i.e. EPS-3688 and EPS-3707 solutions) appeared very different from those observed in other polysaccharides from algae (alginate [43], fucoidan [44]) or from other microalgae and notably red microalgae (*Porphyridium* sp., *P. aerugineum, Dixoniella grisea* and *Rhodella reticulata* [45,46]), which behave as entangled polymer solutions. Some elastic gel behavior has been evidenced for 2% w/w EPSs from *Porphyridium cruentum* with a high elastic modulus (G’ = 10 Pa) [14]. The peculiar behavior of EPS-3707 and 3688, as shown in Figure 2 and Table 2, consists of the very low values of both the yield stress and elastic moduli that correspond to a very weak and fragile gel.

**Figure 3.**
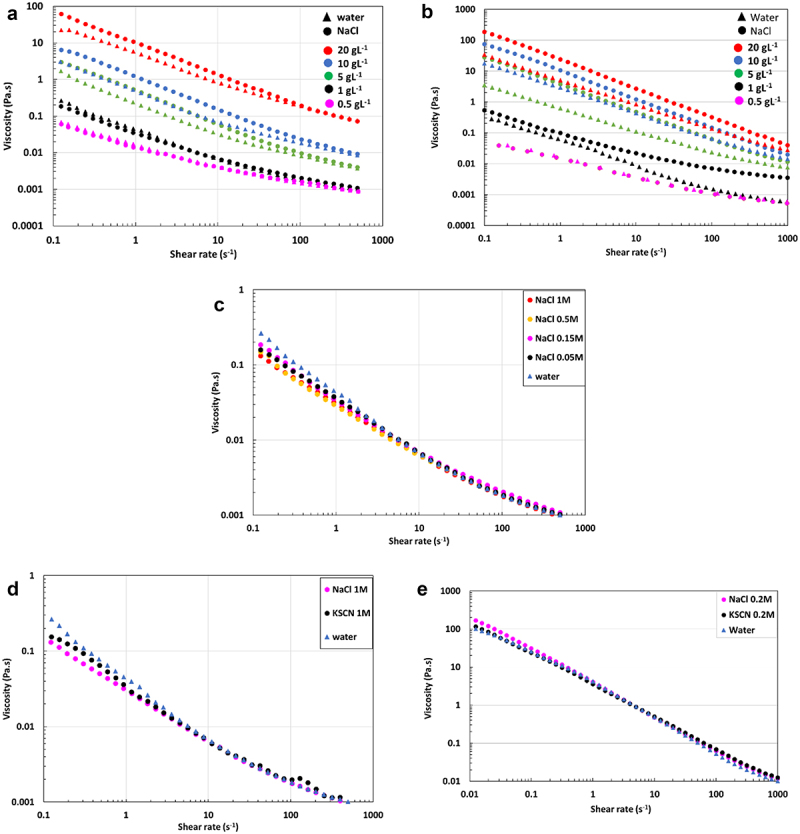
Flow curves : effect of salinity on viscosity of a) EPS-3707 and b) EPS-3688 solutions for various concentrations in water and NaCl 0.15M; c) effect of NaCl concentration on viscosity of EPS-3707 at 1 g L^−1^; d) effect of salt nature on viscosity of EPS-3707 solutions at 1 g L^−1^; e) effect of salt nature on viscosity of EPS-3688 solutions at 5 g L^−1^ in water, KSCN 0.2 mol L^−1^ and NaCl 0.2 mol L^−1^.

EPS solutions at various concentrations have been prepared in NaCl, a lyotropic salt (strengthening hydrophobic interactions [47]), or KSCN, a chaotropic salt (weakening hydrophobic interactions [47]), according to the Hofmeister series to study the impact of salinity on their rheology. The flow curves for the EPS-3707 and EPS-3688 solutions in NaCl 0.15 mol L^−1^ are compared with those in water in Figure 3(a,b), respectively. Regardless of the EPS, the effect of NaCl was more important at higher concentrations (20, 10 and 5 g L^−1^). The presence of NaCl led to the screening of charges present in polysaccharides with a decrease in electrostatic interactions, leading to a possible increase in hydrophobic interactions, causing a gain in viscosity.

The effect on NaCl concentration was also studied on EPS-3707 at 1 g L^−1^ in NaCl 1, 0.5, 0.15 and 0.05 mol L^−1^ Figure 3(c). The variation in NaCl concentration had a low impact on solution viscosities. One can suppose that a low NaCl concentration is sufficient to screen the charges. No effect of KSCN on the viscosity of EPS-3707 1 g L^−1^ and EPS-3688 5 g L^−1^ solutions was detected Figure 3(d,e) respectively.

**Figure 4.**
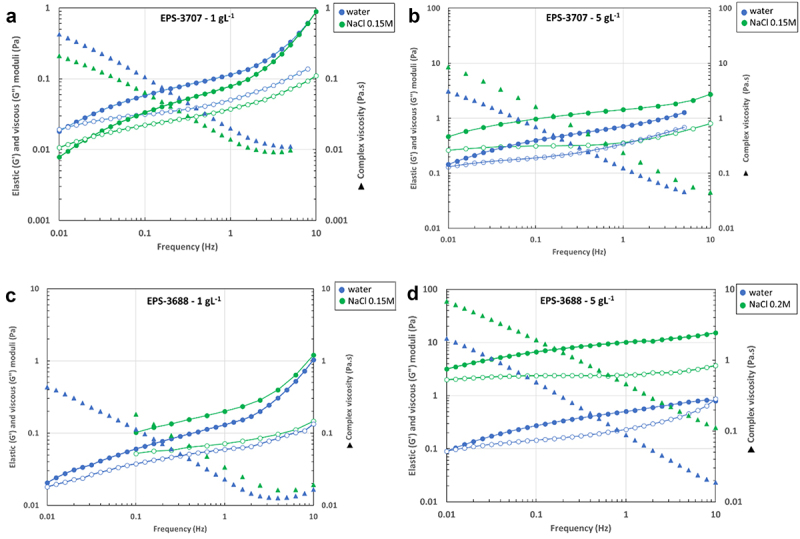
Effect of salinity on oscillation measurements. Left axis: elastic and viscous moduli, right axis: complex viscosity versus frequency of a) EPS-3707 1 g L^−1^ in water and NaCl 0.15 mol L^−1^; b) EPS-3707 5 g L^−1^ in water and NaCl 0.15 mol L^−1^; c) of EPS-3688 1 g L^−1^ in water and NaCl 0.15 mol L^−1^; d) of EPS-3688 5 g L^−1^ in water and NaCl 0.2 mol L^−1^; G’ (fill circle); G’’ (blank circle); complex viscosity (fill triangle).

The impact of salinity on the dynamics of the EPS solutions was examined by oscillation procedures between 0.01 and 10 Hz (the linearity domain of each solution had been checked before) (Figure 4). For EPS-3707 at the lower concentration (i.e., 1 g L^−1^, Figure 4(a), the rheological behavior appeared visco-elastic in both media water and NaCl (0.15 M), but the presence of salt led to a speeding up of the dynamic (relaxation times move from 85 s in water to 25 s in NaCl 0.15 mol L^−1^). At the same time, the modulus value at the cross point did not change (G’ ~ 0.02 Pa), which seems to indicate only a modification of the dynamic. For EPS-3707 at 5 g L^−1^, when the system behaved as a weak gel Figure 4(b), the presence of NaCl 0.15 M led to a significant increase in the elastic behavior, G’ was largely increased, and the dynamics were largely slowed down. The same behavior was also observed for EPS-3688 in 1 and 5 g L^−1^ solutions (Figure 4(c,d), respectively). These observations seem to confirm that ionic strength led to the reinforcement of the three-dimensional structure, probably due to hydrophobic associations.

Comparable behavior has been observed with other anionic and amphiphilic polysaccharides. This is the case in particular with hydrophobically (with stearyl alkyl group) and hydrophilically (with sulfonic-acid salt group) modified hydroxyethylcellulose [48]. The viscosity in the Newtonian domain was increased by the added salt. The authors also observed a significant gain in elastic and viscous moduli. They explained these results as a competition between two antagonist interactions. In the absence of salts, the electrostatic repulsion between the ionic substituents inhibits the hydrophobic aggregations of alkyl substituents and thus leads to the weak formation of the three-dimensional network structure. When salt is added to the modified polysaccharide solution, ionized groups are constricted by cations, and the electrostatic repulsion is weakened. By the charge-screening effects, the strength of hydrophobic associations is predominant in the viscoelasticity of the network structure. These results suggested that the added salt strengthened the three-dimensional network structure by the formation of cross-linkages through the association of hydrophobic substituents.

**Figure 5.**
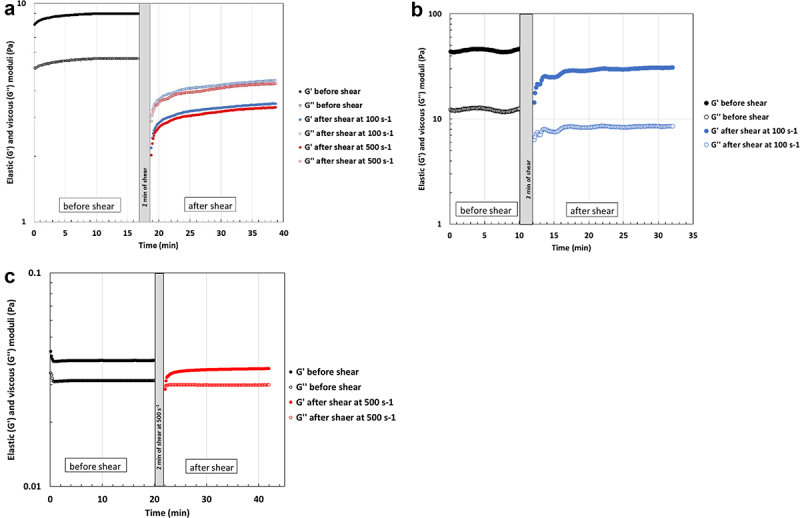
Restructuration of weak gel from EPS-3707 after high shear rate treatment at 25°C. Measurement at 0.1 Hz in the linearity domain a) 20 g L^−1^ in water b) 20 g L^−1^ in NaCl 0.15 mol L^−1^ c) 1 g L^−1^ in NaCl 1 mol L^−1^.

To show that these gels are sensitive to shear but reform at rest, their mechanical properties were measured after an important shear (100 or 500 s^−1^) for 2 min, which destructured them completely. Figure 5 shows a progressive return of modulus values in water and a much faster return in the presence of NaCl. NaCl facilitates this restructuring by screening the negative charges carried by the polysaccharide chain. Indeed, sulfate and significant amounts of uronic acids (glucuronic and galacturonic acids) have been identified as minor monosaccharides in the composition of the two EPSs [22]. At lower concentrations, restructuring was logically more rapid due to the less entanglement between chains and much weaker intermolecular interactions.

### 3.4. Diffusing Wave Spectroscopy (DWS)

Table 4 gives EI at 25, 37, and 60°C for the two EPSs and for alginate (Mw 235 000 g mol^−1^ M/G 1.2) at 1 g L^−1^ in water. Alginate was chosen for its typical behavior in solution without specific polymer/polymer interactions, in contrast to the studied EPSs. At 25°C, the EI values were notably identical for both EPSs and alginate, considering the uncertainty. At higher temperatures, EI was strongly diminished for alginate, which showed an expected loss of elastic characteristics because of the increase in Brownian motion. In contrast, EI remained constant or very slightly diminished for the two EPSs, indicating that the elastic behavior is conserved even at higher temperatures. This is a supplementary indication of the associated structure of the EPS weak gels. It seems that interactions leading to the fragile and weak gel structure were sufficiently strong to not be influenced by temperature. On the other hand, the elasticity was logically reduced with a decrease in the concentration of EPS (Table 5).

**Table 4.** The EI and SLB values of EPSs and alginate solutions in water at 1 g/L in function of temperature.

	EI x 10^4^ (nm^−2^)			SLB		
T (°C)	EPS-3688	EPS-3707	ALG	EPS-3688	EPS-3707	ALG
25	9.7 ± 0.1	9.3 ± 0.3	9.8 ± 1.1	0.53 ± 0.09	0.62 ± 0.08	0.97 ± 0.09
37	10.1 ± 0.7	9.1 ± 0.2	5.7 ± 0.1	0.44 ± 0.06	0.64 ± 0.02	1.04 ± 0.10
60	10.7 ± 0.8	6.8 ± 0.3	3.9 ± 0.1	0.47 ± 0.04	0.73 ± 0.09	0.80 ± 0.12

**Table 5.** The EI values (x 10^4^ (nm^−2^)) of EPS-3688 solutions in water in function of concentration and temperature.

T (°C) C (g L^−1^)	25	37	60
1	9.7 ± 0.1	10.1 ± 0.7	10.7 ± 0.8
0.75	8.9 ± 0.6	8.9 ± 0.6	9.2 ± 0.3
0.5	6.8 ± 0.3	6.6 ± 0.4	7.6 ± 0.6
0.1	5.4 ± 0.1	5.0 ± 0.4	3.7 ± 0.2

The SLBs of EPSs and alginate at 1 g L^−1^ at different temperatures are shown in Table 4. At 25°C, EPSs had an elastic-like behavior, and alginate had clearly a viscous-like behavior. The SLB values confirmed the tendency observed for EI. As an example, the SLB values obtained for EPS-3688 remain quite constant at approximately 0.5 (mainly elastic behavior) regardless of the temperature, whereas SLB values obtained for alginate were clearly increased (toward viscous behavior) when the temperature increased.

**Figure 6.**
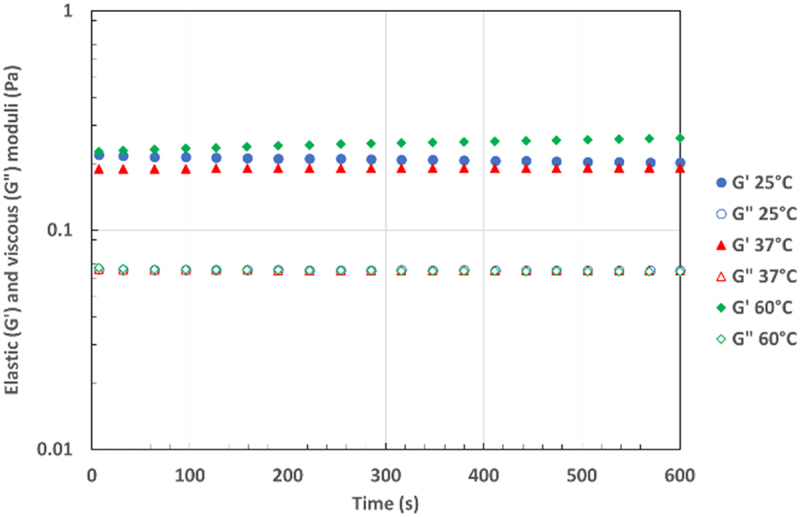
Elastic (G’) and viscous (G’’) moduli versus time at 25°C (blue), 37°C (red) and 60°C (green), of water solution EPS-3688 at 1 g L^−1^, at 1Hz and oscillation stress 0.01Pa.

The temperature influence was checked by classical rheological measurements (time sweep in oscillation at 1 Hz), and the results showed that G’ was quasi-constant at 25, 37, and 60°C (Figure 6) and always higher than G’’ (elastic behavior regardless of the temperature) with a value of tan δ equal to 0.5. Consequently, the elastic-like behavior of EPS solution was not influenced by temperature until 60°C.

In conclusion, this rheological (flow and oscillation) and DWS study clearly shows the atypical behavior of aqueous solutions obtained with the two EPSs produced by *Glossomastix sp*. Even in very dilute media, the solutions exhibit weak gel-like behavior, highly sensitive to shear stress and therefore fragile.

### 3.5. Stability of dispersions

**Figure 7.**
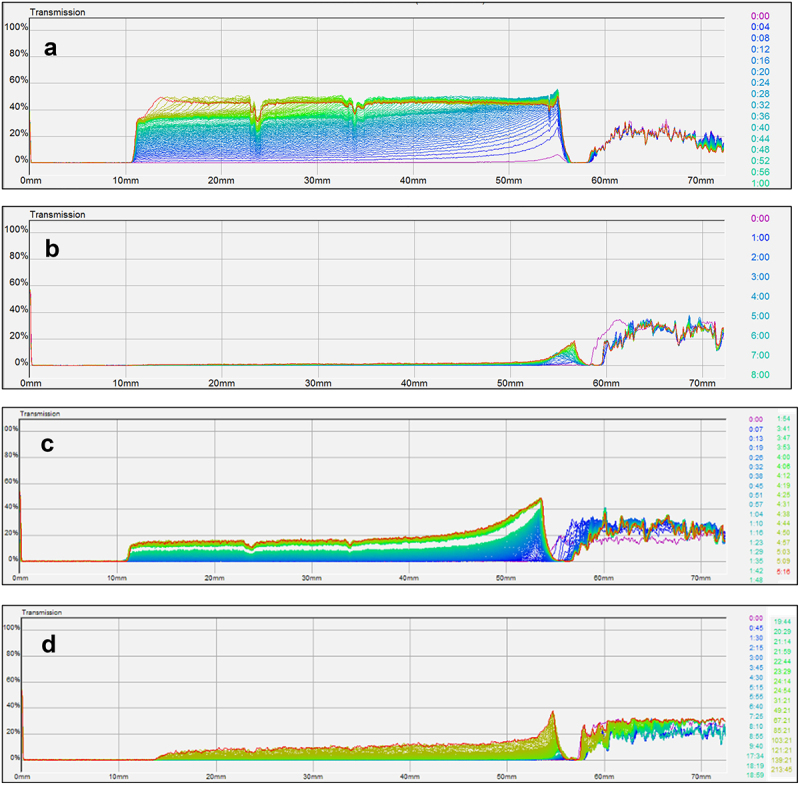
Percentage of transmission (y-axis) as a function of the height of the tube (x-axis) and time (curves from purple at *t*=0 to green and red at t_max_) for a) suspensions of microcrystalline cellulose in pure water; b) suspensions of microcrystalline cellulose in EPS-3688 1 g L^−1^; c) suspensions of microcrystalline cellulose in alginate 10 g L^−1^; d) suspensions of microcrystalline cellulose in EPS-3707 1 g L^−1^.

**Figure 8.**
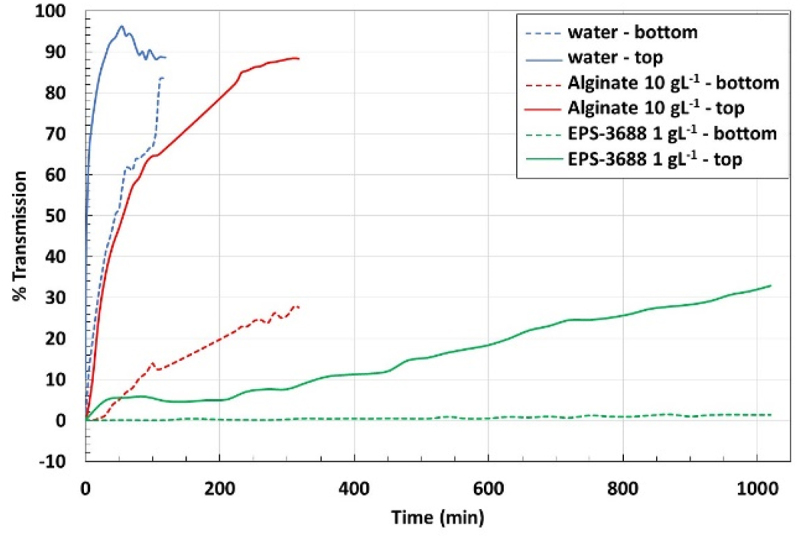
Percentage of transmission with ageing time of suspensions of microcrystalline cellulose.

EPSs could be used as stabilizers of dispersions thanks to their elastic behavior even at low concentrations. The stability of microcrystalline cellulose suspensions was studied in different media (water and alginate/EPSs solutions) using MLS. Figure 7(a) shows an example of graphs obtained for analysis of microcrystalline cellulose suspension in pure water. The x-axis represents the height of the tube of analysis, and the y-axis, the percentage of transmission, and the aging time are represented by the different colors. The transmission increased quickly and reached its maximum in a few minutes. The suspension was very unstable due to rapid sedimentation of particles because of their large size. Figure 7(b,c) were obtained with microcrystalline cellulose suspensions in EPS-3688 (1 g L^−1^) and in alginate (10 g L^−1^) solutions. This concentration of alginate was chosen to have a Newtonian viscosity (0.27 Pa s) comparable to the apparent viscosity of EPS-3688 and EPS-3707 solutions at 1 g L^−1^ for a shear rate of 0.1 s^−1^. The differences between the two media clearly showed the stability of the suspension in EPS-3688 solution (even at 1 g L^−1^). In an alginate solution, the sedimentation was slowed down slightly. In the EPS-3688 solution, the transmission remained constant at all heights of the tube and over the duration of the measurement (approximately 17 h). By plotting the percentage of transmission with aging time in the top (full line) or in the bottom (dotted line) of the tube, the different suspensions can be compared (Figure 8). Thus, it can be seen that the cellulose microcrystalline suspension in EPS-3688 was much more stable than that in alginate solution at 10 g L^−1^. Indeed, the percentage of transmission at the bottom of the tube remained constant, and only a slight increase in transmission was observed at the extreme top of the tube (the maximal value was 32% after 17 h) due to the onset of sedimentation. In contrast, in alginate, the transmission increased from the beginning at the top of the tube (reaching 88% in 5 h30) and more slowly at the bottom of the tube (reaching 27% after 5 h30), confirming the rapid onset of sedimentation. An analysis at longer times (9 days) was carried out with EPS-3707 in the presence of NaCl 0.1 mol L^−1,^ confirming the effect of delay on sedimentation Figure 7(d)

EPSs from *Glossomastic*s sp. are therefore good stabilizers of suspensions. Weak gel behavior can greatly slow down (several days) particle sedimentation. This time will depend on several factors such as particle size, dispersity, density in accordance with the Stockes law [49].

## 4. Conclusion

The search for new sources of polysaccharides is an important issue for manufacturers, in line with the circular bioeconomy. *Glossomastix* sp. produced and secreted rhamnofucan (fucose (40–56%) and rhamnose (20–31%) with complex structures and very high molecular weights as EPSs. These polysaccharides exhibit in aqueous solutions, high molar masses, and aggregated structures in dilute regime that strongly modify the rheological properties even at low concentrations. All the studied solutions, whether in the absence or presence of salt, showed strong rheofluidity and yield stress behaviors. The low values of the yield stresses (between 0.01 and 2.3 Pa depending on the EPS concentration from 0.5 to 10 g L^−1^) together with very low elastic moduli (between 0.01 and 5 Pa at 1 Hz depending on the EPS concentration from 0.5 to 10 g L^−1^) are characteristic of original organized structures with hydrophobic interactions that correspond to a weak and fragile physical gel. The gel texture was therefore very sensitive to stress and then reformed over time. Such systems allow stabilization of dispersions, as with microcrystalline cellulose, a model system studied. Sedimentation was markedly slowed even at low EPS concentrations when compared to sedimentation in a concentrated alginate solution. These results open interesting ways to stabilization of food beverage or cosmetic innovative formulations for instance [5,8]. Further studies are needed on other microalgae to find a relationship between structure and activity.

## Supplementary Material

dulong et al supplementary information.docx

## Data Availability

The authors confirm that the data supporting the findings of this study are available within the article and its supplementary materials, and may be shared upon request.
