# Supplementary material for: Exopolysaccharide from marine microalgae belonging to the Glossomastix genus: fragile gel behavior and suspension stability
Source: Bioengineered. 2023 Dec 28;15(1):2296257. doi: 10.1080/21655979.2023.2296257 (PMC10761178; doi:10.1080/21655979.2023.2296257)
Supplement: dulong et al supplementary information.docx [file KBIE_A_2296257_SM1341.docx]

**Exopolysaccharide from marine microalgae belonging to *Glossomastix* genus: fragile gel behavior and suspension stability**

Virginie Dulong ^a^, Christophe Rihouey ^a^, Clément Gaignard ^b^, Nicolas Bridiau ^c^, Priscilla Gourvil ^d^, Céline Laroche ^b^, Guillaume Pierre ^b^, Tony Varacavoudin ^a^, Ian Probert ^d^, Thierry Maugard ^c^, Philippe Michaud ^b^, Luc Picton ^a^, Didier Le Cerf ^a *^

^a^ Université de Rouen Normandie, INSA Rouen Normandie, CNRS, PBS UMR 6270, F-76000 Rouen, France

^b^ Université Clermont Auvergne, Clermont Auvergne INP, CNRS, Institut Pascal, F-63000 Clermont-Ferrand, France

^c^ La Rochelle Université, CNRS, LIENSs, UMR 7266, F-17000, La Rochelle, France

^d^ Station Biologique de Roscoff (SBR), FR2424, Sorbonne Université, CNRS, Place Georges Teissier, 29680 Roscoff, France

Table S1 : Molecular identification of the typical structures found in the EPS-3707

| **t_R_** | **Ion (m/z)** | | | **Ionized adduct** | **Adduct molecular formula** | **Candidate** | | |
| --- | --- | --- | --- | --- | --- | --- | --- | --- |
|  | ***Experimental value*** | ***Predicted value*** | **Resolution (ppm)** |  |  | ***Composition **** | ***DP*** | **Sulfate groups** |
| 1.01 | *339.0932* | *339.0927* | 1.47 | [M-H]^-^ | [C_12_H_21_O_11_]^-^ | **GalA/GlcA + Rha/Fuc** | **2** | **0** |
| 1.01 | *679.1935* | *679.1932* | 0.44 | [2M-H]^-^ | [C_24_H_39_O_22_]^-^ | **GalA/GlcA + Rha/Fuc** | **2** | **0** |
| 1.45 | *243.0178* | *243.0175* | 1.23 | [M-H]^-^ | [C_6_H_11_O_8_S]^-^ | **Rha/Fuc** | **1** | **0** |
| 1.45 | *487.0431* | *487.0428* | 0.62 | [2M-H]^-^ | [C_12_H_23_O_16_S_2_]^-^ | **Rha/Fuc** | **1** | **0** |
| 1.53 | *535.134* | *535.1333* | 1.31 | [M-H]^-^ | [C_18_H_31_O_16_S]^-^ | **3Rha/Fuc** | **3** | **1** |
| 1.62 | *681.1931* | *681.1912* | 2.79 | [M-H]^-^ | [C_24_H_41_O_20_S]^-^ | **4Rha/Fuc** | **4** | **1** |
| 1.73 | *827.2505* | *827.2491* | 1.69 | [M-H]^-^ | [C_30_H_51_O_24_S]^-^ | **5Rha/Fuc** | **5** | **1** |
| 2.03 | *973.3087* | *973.307* | 1.75 | [M-H]^-^ | [C_36_H_61_O_28_S]^-^ | **6Rha/Fuc** | **6** | **1** |
| 2.16 | *1119.38* | *1119.3649* | 13.49 | [M-H]^-^ | [C_42_H_71_O_32_S]^-^ | **7Rha/Fuc** | **7** | **1** |
| 3.64 | *681.192* | *681.1912* | 1.17 | [M-H]^-^ | [C_24_H_41_O_20_S]^-^ | **4Rha/Fuc** | **4** | **1** |
| 4.81 | *565.1079* | *565.1075* | 0.71 | [M-H]^-^ | [C_18_H_29_O_18_S]^-^ | **2Rha/Fuc + GalA/GlcA** | **3** | **1** |
| 5.33 | *565.1079* | *565.1075* | 0.71 | [M-H]^-^ | [C_18_H_29_O_18_S]^-^ | **2Rha/Fuc + GalA/GlcA** | **3** | **1** |
| 5.33 | *1003.2813* | *1003.2812* | 0.1 | [M-H]^-^ | [C_36_H_59_O_30_S]^-^ | **5Rha/Fuc + GalA/GlcA** | **5** | **1** |
| 5.33 | *1149.3394* | *1149.3391* | 0.26 | [M-H]^-^ | [C_42_H_69_O_34_S]^-^ | **6Rha/Fuc + GalA/GlcA** | **6** | **1** |
| 5.69 | *419.0493* | *419.0496* | 0.72 | [M-H]^-^ | [C_12_H_19_O_14_S]^-^ | **Rha/Fuc + GalA/GlcA** | **2** | **1** |
| 5.69 | *857.2249* | *857.2233* | 1.87 | [M-H]^-^ | [C_30_H_49_O_26_S]^-^ | **4Rha/Fuc + GalA/GlcA** | **6** | **1** |
| 6.11 | *419.0493* | *419.0496* | 0.72 | [M-H]^-^ | [C_12_H_19_O_14_S]^-^ | **Rha/Fuc + GalA/GlcA** | **2** | **1** |
| 6.11 | *565.1079* | *565.1075* | 0.71 | [M-H]^-^ | [C_18_H_29_O_18_S]^-^ | **2Rha/Fuc + GalA/GlcA** | **3** | **1** |
| 6.11 | *857.2227* | *857.2233* | 0.7 | [M-H]^-^ | [C_30_H_49_O_26_S]^-^ | **4Rha/Fuc + GalA/GlcA** | **5** | **1** |
| 6.55 | *565.108* | *565.1075* | 0.88 | [M-H]^-^ | [C_18_H_29_O_18_S]^-^ | **2Rha/Fuc + GalA/GlcA** | **3** | **1** |
| 6.76 | *681.1931* | *681.1912* | 2.79 | [M-H]^-^ | [C_24_H_41_O_20_S]^-^ | **4Rha/Fuc** | **4** | **1** |
| 6.76 | *380.0708* | *380.0701* | 1.84 | [M-2H]^2-^ | [C_24_H_41_O_23_S_2_]^-^ | **4Rha/Fuc** | **4** | **2** |
| 6.76 | *761.1482* | *761.148* | 0.26 | [M-H]^-^ | [C_24_H_41_O_23_S_2_]^-^ | **4Rha/Fuc** | **4** | **2** |
| 6.76 | *876.2846* | *876.2841* | 0.57 | [M-H]^-^ | [C_31_H_58_NO_23_S_2_]^-^ | **4Rha/Fuc** | **4** | **2** |
| 7.16 | *827.2501* | *827.2491* | 1.21 | [M-H]^-^ | [C_30_H_51_O_24_S]^-^ | **5Rha/Fuc** | **5** | **1** |
| 7.16 | *453.0996* | *453.099* | 1.32 | [M-2H]^2-^ | [C_24_H_41_O_23_S_2_]^-^ | **5Rha/Fuc** | **5** | **2** |
| 7.16 | *907.2065* | *907.2059* | 0.66 | [M-H]^-^ | [C_30_H_51_O_27_S_2_]^-^ | **5Rha/Fuc** | **5** | **2** |
| 7.16 | *929.1889* | *929.1879* | 1.08 | [M+Na-2H]^-^ |    \| [C_30_H_50_O_27_S_2_Na]^-^ \| \| --- \| | **5Rha/Fuc** | **5** | **2** |
| 7.16 | *1022.3424* | *1022.342* | 0.39 | [M+C_7_H_15_NH_3_-2H]^-^ | [C_37_H_68_NO_27_S_2_]^-^ |    \| **5Rha/Fuc** \| \| --- \| |    \| **5** \| \| --- \| |    \| **2** \| \| --- \| |
| 7.56 | *535.134* | *535.1333* | 1.31 | [M-H]^-^ | [C_18_H_31_O_16_S]^-^ |    \| **3Rha/Fuc** \| \| --- \| |    \| **3** \| \| --- \| |    \| **1** \| \| --- \| |
| 7.56 | *615.0909* | *615.0901* | 1.3 | [M-H]^-^ | [C_18_H_31_O_19_S_2_]^-^ |    \| **3Rha/Fuc** \| \| --- \| | \| **3** \| \| --- \| | \| **2** \| \| --- \| |
| 7.56 | *637.0724* | *637.072* | 0.63 | [M+Na-2H]^-^ | [C_18_H_30_O_19_S_2_Na]^-^ | \| **3Rha/Fuc** \| \| --- \| | \| **5** \| \| --- \| | \| **2** \| \| --- \| |
| 7.56 | *730.2267* | *730.2262* | 0.68 | [M+C_7_H_15_NH_3_-2H]^-^ | [C_25_H_48_NO_19_S_2_]^-^ | **3Rha/Fuc** | **3** | **2** |
| 8.08 | *681.1916* | *681.1912* | 0.59 | [M-H]- | [C_24_H_41_O_20_S]^-^ | **4Rha/Fuc** | **4** | **1** |
| 8.08 | *761.1478* | *761.148* | 0.26 | [M-H]^-^ | [C_24_H_41_O_23_S_2_]^-^ | **4Rha/Fuc** | **4** | **2** |
| 8.08 | *783.1299* | *783.1299* | 0 | [M+Na-2H]^-^ | [C_24_H_40_O_23_S_2_Na]^-^ | **4Rha/Fuc** | **4** | **2** |
| 8.11 | *491.1332* | *491.1325* | 1.43 | [M-2H]^2-^ | [C_36_H_54_O_31_]^2-^ | **3Rha/Fuc + 3GalA/GlcA** | **6** | **0** |
| 8.11 | *983.2736* | *983.2727* | 0.92 | [M-H]^-^ | [C_36_H_55_O_31_]^-^ | **3Rha/Fuc + 3GalA/GlcA** | **6** | **0** |
| 8.45 | *516.1239* | *516.1238* | 0.19 | [M-2H]^2-^ | [C_36_H_56_O_32_S]^2-^ | **4Rha/Fuc + 2GalA/GlcA** | **6** | **1** |
| 8.45 | *1033.2551* | *1033.2554* | 0.29 | [M-H]^-^ | [C_36_H_57_O_32_S]^-^ | **4Rha/Fuc + 2GalA/GlcA** | **6** | **1** |
| 8.64 | *837.2154* | *837.2148* | 0.72 | [M-H]^-^ | [C_30_H_45_O_27_]^-^ | **2Rha/Fuc + 3GalA/GlcA** | **5** | **0** |
| 8.74 | *516.1239* | *516.1238* | 0.19 | [M-2H]^2-^ | [C_36_H_56_O_32_S]^2-^ | **4Rha/Fuc + 2GalA/GlcA** | **6** | **1** |
| 8.74 | *1033.2551* | *1033.2554* | 0.29 | [M-H]^-^ | [C_36_H_57_O_32_S]^-^ | **4Rha/Fuc + 2GalA/GlcA** | **6** | **1** |
| 9.47 | *516.1239* | *516.1238* | 0.19 | [M-2H]^2-^ | [C_36_H_56_O_32_S]^2-^ | **4Rha/Fuc + 2GalA/GlcA** | **6** | **1** |
| 9.47 | *1033.2551* | *1033.2554* | 0.29 | [M-H]^-^ | [C_36_H_57_O_32_S]^-^ | **4Rha/Fuc + 2GalA/GlcA** | **6** | **1** |
| 9.99 | *887.1984* | *887.1975* | 1.01 | [M-H]^-^ | [C_30_H_47_O_28_S]^-^ | **3Rha/Fuc + 2GalA/GlcA** | **5** | **1** |
| 10.17 | *595.082* | *595.0816* | 0.67 | [M-H]^-^ | [C_18_H_27_O_20_S]^-^ | **Rha/Fuc + 2GalA/GlcA** | **3** | **1** |
| 10.17 | *741.1393* | *741.1395* | 0.27 | [M-H]^-^ | [C_24_H_37_O_24_S]^-^ | **2Rha/Fuc + 2GalA/GlcA** | **4** | **1** |
| 10.17 | *887.1984* | *887.1975* | 1.01 | [M-H]^-^ | [C_30_H_47_O_28_S]^-^ | **3Rha/Fuc + 2GalA/GlcA** | **5** | **1** |
| 10.3 | *757.1347* | *757.1345* | 0.26 | [M-H]^-^ | [C_24_H_37_O_25_S]^-^ | **Rha/Fuc + 2GalA/GlcA + Gal/Glc** | **4** | **1** |
| 10.46 | *869.1889* | *869.1869* | 2.3 | [M-H]^-^ | [C_30_H_45_O_27_S]^-^ | **3Rha/Fuc + 2GalA/GlcA** | **5** | **1** |
| 10.56 | *468.0864* | *468.0861* | 0.64 | [M-2H]^2-^ | \| [C_30_H_48_O_29_S_2_]^2-^ \| \| --- \| | **4Rha/Fuc + GalA/GlcA** | **5** | **2** |
| 10.56 | *937.1807* | *937.1801* | 0.64 | [M-H]^-^ | [C_30_H_49_O_29_S_2_]^-^ | \| **4Rha/Fuc + GalA/GlcA** \| \| --- \| | \| **5** \| \| --- \| | \| **2** \| \| --- \| |
| 10.56 | *1052.3174* | *1052.3162* | 1.14 | [M+C_7_H_15_NH_3_-2H]^-^ | [C_37_H_66_NO_29_S_2_]^-^ | \| **4Rha/Fuc + GalA/GlcA** \| \| --- \| | \| **5** \| \| --- \| | \| **1** \| \| --- \| |
| 10.59 | *857.2249* | *857.2233* | 1.87 | [M-H]^-^ | [C_30_H_49_O_26_S]^-^ | \| **4Rha/Fuc + GalA/GlcA** \| \| --- \| | \| **5** \| \| --- \| | \| **1** \| \| --- \| |
| 10.71 | *711.1663* | *711.1654* | 1.27 | [M-H]^-^ | [C_24_H_39_O_22_S]^-^ | \| **3Rha/Fuc + GalA/GlcA** \| \| --- \| | \| **3** \| \| --- \| | \| **1** \| \| --- \| |
| 10.71 | *395.0577* | *395.0572* | 1.27 | [M-H]^-^ | [C_24_H_39_O_25_S_2_]^-^ | **3Rha/Fuc + GalA/GlcA** | **3** | **2** |
| 10.71 | *791.1227* | *791.1222* | 0.63 | [M-H]^-^ | [C_24_H_39_O_25_S_2_]^-^ | **3Rha/Fuc + GalA/GlcA** | **3** | **2** |
| 10.71 | *906.2592* | *906.2583* | 0.99 | [M+C_7_H_15_NH_3_-2H]^-^ | [C_31_H_56_NO_25_S_2_]^-^ | **3Rha/Fuc + GalA/GlcA** | **3** | **2** |
| 10.89 | *545.0986* | *545.099* | 0.73 | [M-H]^-^ | [C_18_H_25_O_19_]^-^ | **3GalA/GlcA** | **3** | **0** |
| 11 | *660.1755* | *660.1749* | 0.91 | [M-2H]^2-^ | [C_48_H_72_O_42_]^2-^ | **3Rha/Fuc + 4GalA/GlcA + Gal/Glc** | **8** | **0** |
| 11 | *1321.3571* | *1321.3576* | 0.38 | [M-H]^-^ | [C_48_H_73_O_42_]^-^ | **3Rha/Fuc + 4GalA/GlcA + Gal/Glc** | **8** | **0** |
| 11.11 | *1159.3059* | *1159.3048* | 0.95 | [M-H]^-^ | [C_42_H_63_O_37_]^-^ | **3Rha/Fuc + 4GalA/GlcA** | **7** | **0** |
| 11.17 | *565.1075* | *565.1075* | 0 | [M-H]^-^ | [C_18_H_29_O_18_S]^-^ | **2Rha/Fuc + GalA/GlcA** | **3** | **1** |
| 11.17 | *645.0652* | *645.0643* | 1.4 | [M-H]^-^ | [C_18_H_29_O_21_S_2_]^-^ | **2Rha/Fuc + GalA/GlcA** | **3** | **2** |
| 11.17 | *760.201* | *760.2004* | 0.79 | [M+C_7_H_15_NH_3_-2H]^-^ | [C_25_H_46_NO_21_S_2_]^-^ | **2Rha/Fuc + GalA/GlcA** | **3** | **2** |
| 11.17 | *1149.339* | *1149.3391* | 0.09 | [M-H]^-^ | [C_42_H_69_O_34_S]^-^ | **6Rha/Fuc + GalA/GlcA** | **7** | **1** |
| 11.17 | *1295.3966* | *1295.397* | 0.31 | [M-H]^-^ | [C_48_H_79_O_38_S]^-^ | **7Rha/Fuc + GalA/GlcA** | **8** | **1** |
| 11.17 | *1355.3448* | *1355.3454* | 0.44 | [M-H]^-^ | [C_48_H_75_O_42_S]^-^ | **5Rha/Fuc + 3GalA/GlcA** | **8** | **1** |
| 11.17 | *1441.45* | *1441.4549* | 3.4 | [M-H]^-^ | [C_54_H_89_O_42_S]^-^ | **8Rha/Fuc + GalA/GlcA** | **8** | **1** |
| 11.17 | *1501.3969* | *1501.4033* | 4.26 | [M-H]^-^ | [C_54_H_85_O_46_S]^-^ | **6Rha/Fuc + 3GalA/GlcA** | **9** | **1** |
| 11.26 | *1003.2813* | *1003.2812* | 0.1 | [M-H]^-^ | [C_36_H_59_O_30_S]^-^ | **5Rha/Fuc + GalA/GlcA** | **6** | **1** |
| 11.26 | *1063.2308* | *1063.2295* | 1.22 | [M-H]^-^ | [C_36_H_55_O_34_S]^-^ | **3Rha/Fuc + 3GalA/GlcA** | **6** | **1** |
| 11.26 | *1149.3413* | *1149.3391* | 1.91 | [M^-^H]^-^ | [C_42_H_69_O_34_S]^-^ | **6Rha/Fuc + GalA/GlcA** | **7** | **1** |
| 11.26 | *1198.3754* | *1198.3741* | 1.08 | [M+C_7_H_15_NH_3_-2H]^-^ | [C_43_H_76_NO_33_S_2_]^-^ | **5Rha/Fuc + GalA/GlcA** | **6** | **2** |
| 11.26 | *1209.2869* | *1209.2875* | 0.5 | [M^-^H]^-^ | [C_42_H_65_O_38_S]^-^ | **4Rha/Fuc + 3GalA/GlcA** | **7** | **1** |
| 11.26 | *1344.4313* | *1344.432* | 0.52 | [M+C_7_H_15_NH_3_-2H]^-^ | \| [C_49_H_86_NO_37_S_2_]^-^ \| \| --- \| | **6Rha/Fuc + GalA/GlcA** | **7** | **2** |
| 11.43 | *458.0818* | *458.0819* | 0.22 | [M-2H]^2-^ | [C_30_H_45_O_30_S]^-^ | \| **2Rha/Fuc + 3GalA/GlcA** \| \| --- \| | \| **5** \| \| --- \| | \| **1** \| \| --- \| |
| 11.43 | *917.1721* | *917.1716* | 0.55 | [M-H]^-^ | [C_30_H_45_O_30_S]^-^ | \| **2Rha/Fuc + 3GalA/GlcA** \| \| --- \| | \| **5** \| \| --- \| | \| **1** \| \| --- \| |
| 11.43 | *1052.3162* | *1052.3162* | 0 | [M+C_7_H_15_NH_3_-2H]^-^ | [C_37_H_66_NO_29_S_2_]^-^ | \| **4Rha/Fuc + GalA/GlcA** \| \| --- \| | \| **5** \| \| --- \| | \| **2** \| \| --- \| |
| 11.43 | *1149.3413* | *1149.3391* | 1.91 | [M-H]^-^ | [C_42_H_69_O_34_S]^-^ | \| **6Rha/Fuc + GalA/GlcA** \| \| --- \| | \| **7** \| \| --- \| | \| **1** \| \| --- \| |
| 11.43 | *614.1445* | *614.144* | 0.81 | [M-2H]^2-^ | [C_42_H_69_O_37_S_2_]^-^ | **6Rha/Fuc + GalA/GlcA** | **7** | **2** |
| 11.43 | *1229.2952* | *1229.2959* | 0.57 | [M-H]^-^ | [C_42_H_69_O_37_S_2_]^-^ | **6Rha/Fuc + GalA/GlcA** | **7** | **2** |
| 11.43 | *1251.2777* | *1251.2779* | 0.16 | [M+Na-2H]^-^ | [C_42_H_68_O_37_S_2_Na]^-^ | **6Rha/Fuc + GalA/GlcA** | **7** | **2** |
| 11.43 | *1344.4313* | *1344.4302* | 0.82 | [M+C_7_H_15_NH_3_-2H]^-^ | [C_49_H_87_NO_37_S_2_]^-^ | **6Rha/Fuc + GalA/GlcA** | **7** | **2** |
| 11.54 | *541.116* | *541.1151* | 1.66 | [M-2H]^2-^ | [C_36_H_58_O_33_S_2_]_2_^-^ | **5Rha/Fuc + GalA/GlcA** | **6** | **2** |
| 11.54 | *1083.2382* | *1083.238* | 0.18 | [M-H]^-^ | [C_36_H_59_O_33_S_2_]^-^ | **5Rha/Fuc + GalA/GlcA** | **6** | **2** |
| 11.54 | *1105.2202* | *1105.2199* | 0.27 | [M+Na-2H]^-^ | [C_36_H_58_O_33_S_2_Na]^-^ | **5Rha/Fuc + GalA/GlcA** | **6** | **2** |
| 11.54 | *629.1317* | *629.1311* | 0.95 | [M-2H]^2-^ | [C_42_H_66_O_39_S_2_]_2_^-^ | **5Rha/Fuc + 2GalA/GlcA** | **7** | **2** |
| 11.54 | *1259.2698* | *1259.2735* | 2.94 | [M-H]^-^ | [C_42_H_67_O_39_S_2_]^-^ | **5Rha/Fuc + 2GalA/GlcA** | **7** | **2** |
| 11.54 | *1281.25* | *1281.252* | 1.56 | [M+Na-2H]^-^ | [C_42_H_67_O_39_S_2_Na]^-^ | **5Rha/Fuc + 2GalA/GlcA** | **7** | **2** |
| 11.54 | *1063.2296* | *1063.2295* | 0.09 | [M-H]^-^ | [C_36_H_55_O_34_S]^-^ | **3Rha/Fuc + 3GalA/GlcA** | **6** | **1** |
| 11.54 | *1159.3044* | *1159.3048* | 0.35 | [M-H]^-^ | [C_42_H_63_O_34_]^-^ | **3Rha/Fuc + 4GalA/GlcA** | **7** | **0** |
| 11.54 | *1198.3754* | *1198.3741* | 1.08 | [M+C_7_H_15_NH_3_-2H]^-^ | [C_43_H_76_NO_33_S_2_]^-^ | **5Rha/Fuc + GalA/GlcA** | **6** | **2** |
| 11.54 | *1374.4038* | *1374.4062* | 1.75 | [M+C_7_H_15_NH_3_-2H]^-^ | [C_49_H_84_NO_39_S_2_]^-^ | **5Rha/Fuc + 2GalA/GlcA** | **7** | **2** |
| 11.67 | *531.1111* | *531.1109* | 0.38 | [M-2H]^2-^ | [C_36_H_54_O_34_S]^2-^ | **3Rha/Fuc + 3GalA/GlcA** | **6** | **1** |
| 11.67 | *1063.229* | *1063.2295* | 0.47 | [M-H]^-^ | [C_36_H_55_O_34_S]^-^ | **3Rha/Fuc + 3GalA/GlcA** | **6** | **1** |
| 11.67 | *1085.2114* | *1085.2115* | 0.09 | [M+Na-2H]^-^ | [C_36_H_54_O_34_NaS]^-^ | **3Rha/Fuc + 3GalA/GlcA** | **6** | **1** |
| 11.76 | *1033.2563* | *1033.2554* | 0.87 | [M-H]^-^ | [C_36_H_57_O_32_S]^-^ | **4Rha/Fuc + 2GalA/GlcA** | **6** | **1** |
| 11.76 | *556.1033* | *556.1022* | 1.98 | [M-2H]^2-^ | [C_36_H_56_O_35_S_2_]^2-^ | **4Rha/Fuc + 2GalA/GlcA** | **6** | **2** |
| 11.76 | *1113.2129* | *1113.2122* | 0.63 | [M-H]^-^ | [C_36_H_57_O_35_S_2_]^-^ | **4Rha/Fuc + 2GalA/GlcA** | **6** | **2** |
| 11.76 | *1135.1953* | *1135.1941* | 1.06 | [M+Na-2H]^-^ | [C_36_H_56_O_35_S_2_Na]^-^ | **4Rha/Fuc + 2GalA/GlcA** | **6** | **2** |
| 11.76 | *1228.3489* | *1228.3483* | 0.49 | [M+C_7_H_15_NH_3_-2H]^-^ | [C_43_H_74_NO_35_S_2_]^-^ | **4Rha/Fuc + 2GalA/GlcA** | **6** | **2** |
| 11.98 | *700.1533* | *700.1533* | 0 | [M-2H]^2-^ | [C_48_H_72_O_45_S]^2-^ | **3Rha/Fuc + 4GalA/GlcA + Gal/Glc** | **8** | **1** |
| 11.98 | *1401.3083* | *1401.3145* | 4.42 | [M-H]^-^ | [C_48_H_73_O_45_S]^-^ | **3Rha/Fuc + 4GalA/GlcA + Gal/Glc** | **8** | **1** |
| 12.16 | *773.1838* | *773.1823* | 1.94 | [M-2H]^2-^ | [C_52_H_82_O_49_S]^2-^ | **4Rha/Fuc + 4GalA/GlcA + Gal/Glc** | **9** | **1** |
| 12.16 | *1547.3589* | *1547.3724* | 8.72 | [M-H]^-^ | [C_52_H_83_O_49_S]^-^ | **4Rha/Fuc + 4GalA/GlcA + Gal/Glc** | **9** | **1** |
| 12.19 | *887.1984* | *887.1975* | 1.01 | [M-H]^-^ | [C_30_H_47_O_28_S]^-^ | **3Rha/Fuc + 2GalA/GlcA** | **5** | **1** |
| 12.19 | *483.074* | *483.0732* | 1.66 | [M-2H]^2-^ | [C_30_H_46_O_31_S_2_]^2-^ | **3Rha/Fuc + 2GalA/GlcA** | **5** | **2** |
| 12.19 | *967.1552* | *967.1543* | 0.93 | [M-H]^-^ | [C_30_H_47_O_31_S_2_]^-^ | **3Rha/Fuc + 2GalA/GlcA** | **5** | **2** |
| 12.19 | *989.1376* | *989.1362* | 1.42 | [M+Na-2H]^-^ | [C_30_H_46_O_31_S_2_Na]^-^ | **3Rha/Fuc + 2GalA/GlcA** | **5** | **2** |
| 12.19 | *1082.2921* | *1082.2904* | 1.57 | [M-H]^-^ | [C_37_H_64_NO_31_S_2_]^-^ | **3Rha/Fuc + 2GalA/GlcA** | **5** | **2** |
| 12.19 | *692.1568* | *692.1559* | 1.3 | [M-2H]^2-^ | [C_48_H_72_O_44_S]^2-^ | **4Rha/Fuc + 4GalA/GlcA** | **8** | **1** |
| 12.19 | *1385.3191* | *1385.3195* | 0.29 | [M-H]^-^ | [C_48_H_73_O_44_S]^-^ | **4Rha/Fuc + 4GalA/GlcA** | **8** | **1** |
| 12.19 | *790.1776* | *790.1761* | 1.9 | [M-2H]^2-^ | [C_54_H_84_O_49_S_2_]^2-^ | **6Rha/Fuc + 3GalA/GlcA** | **9** | **2** |
| 12.19 | *1581.3658* | *1581.3601* | 3.6 | [M-H]^-^ | [C_54_H_85_O_49_S_2_]^-^ | **6Rha/Fuc + 3GalA/GlcA** | **9** | **2** |
| 12.33 | *692.1568* | *692.1559* | 1.3 | [M-2H]^2-^ | [C_48_H_72_O_44_S]^2-^ | **4Rha/Fuc + 4GalA/GlcA** | **8** | **1** |
| 12.33 | *1385.3191* | *1385.3195* | 0.29 | [M-H]^-^ | [C_48_H_73_O_44_S]^-^ | **4Rha/Fuc + 4GalA/GlcA** | **8** | **1** |
| 12.33 | *790.1776* | *790.1761* | 1.9 | [M-2H]^2-^ | [C_54_H_84_O_49_S_2_]^2-^ | **6Rha/Fuc + 3GalA/GlcA** | **9** | **2** |
| 12.33 | *1581.3658* | *1581.3601* | 3.6 | [M-H]^-^ | [C_54_H_85_O_49_S_2_]^-^ | **6Rha/Fuc + 3GalA/GlcA** | **9** | **2** |
| 12.45 | *483.074* | *483.0732* | 1.66 | [M-2H]^2-^ | [C_30_H_46_O_31_S_2_]^2-^ | **3Rha/Fuc + 2GalA/GlcA** | **5** | **2** |
| 12.45 | *967.1552* | *967.1543* | 0.93 | [M-H]^-^ | [C_30_H_47_O_31_S_2_]^-^ | **3Rha/Fuc + 2GalA/GlcA** | **5** | **2** |
| 12.45 | *700.1533* | *700.1533* | 0 | [M-2H]^2-^ | [C_48_H_72_O_45_S]^2-^ | **3Rha/Fuc + 4GalA/GlcA + Gal/Glc** | **8** | **1** |
| 12.45 | *1401.3083* | *1401.3145* | 4.42 | [M-H]^-^ | [C_48_H_73_O_45_S]^-^ | **3Rha/Fuc + 4GalA/GlcA + Gal/Glc** | **8** | **1** |
| 12.45 | *790.1776* | *790.1761* | 1.9 | [M-2H]^2-^ | [C_54_H_84_O_49_S_2_]^2-^ | **6Rha/Fuc + 3GalA/GlcA** | **9** | **2** |
| 12.45 | *1581.3658* | *1581.3601* | 3.6 | [M-H]^-^ | [C_54_H_85_O_49_S_2_]^-^ | **6Rha/Fuc + 3GalA/GlcA** | **9** | **2** |
| 12.59 | *619.127* | *619.1269* | 0.16 | [M-2H]^2-^ | [C_42_H_62_O_40_S]^2-^ | **3Rha/Fuc + 4GalA/GlcA** | **7** | **1** |
| 12.59 | *1239.2616* | *1239.2616* | 0 | [M-H]^-^ | [C_42_H_63_O_40_S]^-^ | **3Rha/Fuc + 4GalA/GlcA** | **7** | **1** |
| 12.59 | *717.1479* | *717.1472* | 0.98 | [M-2H]^2-^ | [C_48_H_74_O_45_S_2_]^2-^ | **5Rha/Fuc + 3GalA/GlcA** | **8** | **1** |
| 12.59 | *1435.2981* | *1435.3022* | 2.86 | [M-H]^-^ | [C_48_H_75_O_45_S_2_]^-^ | **5Rha/Fuc + 3GalA/GlcA** | **8** | **2** |
| 12.59 | *1550.4248* | *1550.4383* | 8.71 | [M+C_7_H_15_NH_3_-2H]^-^ | [C_55_H_92_NO_45_S_2_]^-^ | **5Rha/Fuc + 3GalA/GlcA** | **8** | **1** |
| 12.85 | *644.1194* | *644.1182* | 1.86 | [M-2H]^2-^ | [C_42_H_64_O_41_S_2_]_2_^-^ | **4Rha/Fuc + 3GalA/GlcA** | **7** | **2** |
| 12.85 | *717.1482* | *717.1472* | 1.39 | [M-2H]^2-^ | [C_48_H_74_O_48_S_3_]^2-^ | **5Rha/Fuc + 3GalA/GlcA** | **8** | **2** |
| 12.85 | *1457.2783* | *1457.2841* | 3.98 | [M+Na-2H]^-^ | [C_48_H_74_O_48_S_3_Na]^-^ | **4Rha/Fuc + 3GalA/GlcA** | **8** | **2** |
| 12.85 | *934.2288* | *934.2273* | 1.61 | [M-2H]^2-^ | [C_66_H_101_O_59_S]^2-^ | **5Rha/Fuc + 5GalA/GlcA + Gal/Glc** | **10** | **1** |
| 13.05 | *571.0903* | *571.0893* | 1.75 | [M-2H]^2-^ | [C_36_H_54_O_37_S_2_]^2-^ | **3Rha/Fuc + 3GalA/GlcA** | **6** | **2** |
| 13.05 | *1063.229* | *1063.2295* | 0.47 | [M-H]^-^ | [C_36_H_55_O_34_S]^-^ | **3Rha/Fuc + 3GalA/GlcA** | **6** | **1** |
| 13.05 | *1258.3212* | *1258.3225* | 1.03 | [M+C_7_H_15_NH_3_-2H]^-^ | [C_43_H_73_NO_37_S_2_]^-^ | **3Rha/Fuc + 3GalA/GlcA** | **6** | **2** |
| 13.33 | *498.0606* | *498.0603* | 0.6 | [M-2H]^2-^ | [C_30_H_44_O_33_S_2_]^2-^ | **2Rha/Fuc + 3GalA/GlcA** | **5** | **2** |
| 13.33 | *917.1705* | *917.1716* | 1.2 | [M-H]^-^ | [C_30_H_45_O_30_S]^-^ | **2Rha/Fuc + 3GalA/GlcA** | **5** | **1** |
| 13.33 | *1112.2631* | *1112.2645* | 1.26 | [M+C_7_H_15_NH_3_-2H]^-^ | [C_43_H_73_NO_37_S_2_]^-^ | **2Rha/Fuc + 3GalA/GlcA** | **5** | **2** |
| 13.64 | *629.131* | *629.1311* | 0.16 | [M-2H]^2-^ | [C_42_H_66_O_39_S_2_]^2-^ | **5Rha/Fuc + 2GalA/GlcA** | **7** | **2** |
| 13.64 | *1259.2766* | *1259.2735* | 2.46 | [M-H]^-^ | [C_42_H_67_O_39_S_2_]^-^ | **5Rha/Fuc + 2GalA/GlcA** | **7** | **2** |
| 13.64 | *669.1092* | *669.1095* | 0.45 | [M-2H]^2-^ | [C_42_H_66_O_42_S_3_]^2-^ | **5Rha/Fuc + 2GalA/GlcA** | **7** | **3** |
| 13.64 | *680.0999* | *680.1005* | 0.88 | [M+Na-2H]^-^ | [C_42_H_65_O_42_S_3_Na]^-^ | **5Rha/Fuc + 2GalA/GlcA** | **7** | **3** |
| 13.64 | *726.6771* | *726.6776* | 0.69 | [M+C_7_H_15_NH_3_-3H]^2-^ | [C_49_H_83_NO_42_S_3_]^2-^ | **5Rha/Fuc + 2GalA/GlcA** | **7** | **3** |
| 13.64 | *619.127* | *619.1269* | 0.16 | [M-2H]^2-^ | [C_42_H_62_O_40_S]^2-^ | **3Rha/Fuc + 4GalA/GlcA** | **7** | **1** |
| 13.64 | *1239.259* | *1239.2616* | 2.1 | [M-H]^-^ | [C_42_H_63_O_40_S]^-^ | **3Rha/Fuc + 4GalA/GlcA** | **7** | **1** |
| 13.64 | *1179.3102* | *1179.3133* | 2.63 | [M-H]^-^ | [C_42_H_67_O_36_S]^-^ | **5Rha/Fuc + 2GalA/GlcA** | **7** | **1** |
| 13.64 | *1383.1874* | *1383.1908* | 2.46 | [M-H]^-^ | [C_42_H_65_O_42_S_3_Na_2_]^-^ | **5Rha/Fuc + 2GalA/GlcA** | **7** | **3** |
| 13.64 | *1476.3369* | *1476.3449* | 5.42 | [M+C_7_H_15_NH_3_-2H]^-^ | [C_49_H_83_NO_42_S_3_Na]^-^ | **5Rha/Fuc + 2GalA/GlcA** | **7** | **3** |
| 14.02 | *732.1348* | *732.1343* | 0.68 | [M-2H]^2-^ | [C_48_H_72_O_47_S_2_]^2-^ | **4Rha/Fuc + 4GalA/GlcA** | **8** | **2** |
| 14.02 | *780.1732* | *780.1719* | 1.67 | [M-2H]^2-^ | [C_54_H_80_O_50_S]^2-^ | **4Rha/Fuc + 5GalA/GlcA** | **9** | **1** |
| 14.02 | *878.192* | *878.1922* | 0.23 | [M-2H]^2-^ | [C_60_H_92_O_55_S_2_]^2-^ | **6Rha/Fuc + 4GalA/GlcA** | **10** | **2** |
| 14.02 | *934.2278* | *934.2273* | 0.54 | [M-2H]^2-^ | [C_66_H_100_O_59_S]^2-^ | **5Rha/Fuc + 5GalA/GlcA + Gal/Glc** | **11** | **1** |
| 14.51 | *556.1046* | *556.1022* | 4.32 | [M-2H]^2-^ | [C_36_H_56_O_35_S_2_]^2-^ | **4Rha/Fuc + 2GalA/GlcA** | **6** | **2** |
| 14.51 | *596.0826* | *596.0806* | 3.36 | [M-2H]^2-^ | [C_36_H_56_O_38_S_3_]^2-^ | **4Rha/Fuc + 2GalA/GlcA** | **6** | **3** |
| 14.51 | *607.0737* | *607.0716* | 3.46 | [M+Na-3H]^2-^ | [C_36_H_55_O_38_S_3_Na]^2-^ | **4Rha/Fuc + 2GalA/GlcA** | **6** | **3** |
| 14.51 | *653.6506* | *653.6486* | 3.06 | [M+C_7_H_15_NH_3_-3H]^2-^ | [C_43_H_73_NO_38_S_3_]^2-^ | **4Rha/Fuc + 2GalA/GlcA** | **6** | **3** |
| 14.51 | *732.1359* | *732.1343* | 2.19 | [M-2H]^2-^ | [C_48_H_74_O_47_S_2_]^2-^ | **4Rha/Fuc + 4GalA/GlcA** | **8** | **2** |
| 14.51 | *805.1659* | *805.1632* | 3.35 | [M-2H]^2-^ | [C_54_H_84_O_51_S_2_]^2-^ | **5Rha/Fuc + 4GalA/GlcA** | **9** | **2** |
| 14.51 | *1033.2551* | *1033.2554* | 0.29 | [M-H]^-^ | [C_36_H_57_O_32_S]^-^ | **4Rha/Fuc + 2GalA/GlcA** | **6** | **1** |
| 15.1 | *790.1771* | *790.1761* | 1.27 | [M-2H]^2-^ | [C_54_H_84_O_49_S_2_]^2-^ | **6Rha/Fuc + 3GalA/GlcA** | **9** | **2** |
| 15.1 | *830.1558* | *830.1545* | 1.57 | [M-2H]^2-^ | [C_54_H_84_O_52_S_3_]^2-^ | **6Rha/Fuc + 3GalA/GlcA** | **9** | **3** |
| 15.1 | *841.1466* | *841.1455* | 1.31 | [M+Na-3H]^2-^ | [C_54_H_83_O_52_S_3_Na]^2-^ | **6Rha/Fuc + 3GalA/GlcA** | **9** | **3** |
| 15.1 | *887.7243* | *887.7226* | 1.92 | [M+C_7_H_15_NH_3_-3H]^2-^ | [C_61_H_101_NO_52_S_3_]^2-^ | **6Rha/Fuc + 3GalA/GlcA** | **9** | **3** |
| 15.28 | *556.1033* | *556.1022* | 1.98 | [M-2H]^2-^ | [C_36_H_56_O_35_S_2_]^2-^ | **4Rha/Fuc + 2GalA/GlcA** | **6** | **2** |
| 15.28 | *596.0815* | *596.0806* | 1.51 | [M-2H]^2-^ | [C_36_H_56_O_38_S_3_]_2_^-^ | **4Rha/Fuc + 2GalA/GlcA** | **6** | **3** |
| 15.28 | *607.0727* | *607.0716* | 1.81 | [M+Na-3H]^2-^ | [C_36_H_55_O_38_S_3_Na]^2-^ | **4Rha/Fuc + 2GalA/GlcA** | **6** | **3** |
| 15.28 | *653.6493* | *653.6486* | 1.07 | [M-2H]^2-^ | [C_43_H_73_NO_38_S_3_]^2-^ | **4Rha/Fuc + 2GalA/GlcA** | **6** | **3** |
| 15.28 | *732.1354* | *732.1343* | 1.5 | [M-2H]^2-^ | [C_48_H_74_O_47_S_2_]^2-^ | **4Rha/Fuc + 4GalA/GlcA** | **8** | **2** |
| 15.57 | *732.1354* | *732.1343* | 1.5 | [M-2H]^2-^ | [C_48_H_72_O_47_S_2_]^2-^ | **4Rha/Fuc + 4GalA/GlcA** | **8** | **2** |
| 15.57 | *966.2103* | *966.2082* | 2.17 | [M-2H]^2-^ | [C_66_H_100_O_61_S_2_]^2-^ | **6Rha/Fuc + 5GalA/GlcA** | **11** | **2** |
| 15.57 | *1121.7985* | *1121.7965* | 1.78 | [M+C_7_H_15_NH_3_-3H]^2-^ | [C_79_H_129_NO_66_S_3_]^2-^ | **8Rha/Fuc + 4GalA/GlcA** | **12** | **3** |
| 15.57 | *1194.8262* | *1194.8255* | 0.59 | [M+C_7_H_15_NH_3_-3H]^2-^ | [C_85_H_139_NO_70_S_3_]_2_^-^ | **9Rha/Fuc + 4GalA/GlcA** | **13** | **3** |
| 15.66 | *659.1071* | *659.1053* | 2.73 | [M-2H]^2-^ | [C_42_H_62_O_43_S_2_]^2-^ | **3Rha/Fuc + 4GalA/GlcA** | **7** | **2** |
| 15.66 | *717.1481* | *717.1472* | 1.25 | [M-2H]^2-^ | [C_48_H_74_O_45_S_2_]^2-^ | **5Rha/Fuc + 3GalA/GlcA** | **8** | **2** |
| 15.66 | *893.1812* | *893.1793* | 2.13 | [M-2H]^2-^ | [C_60_H_90_O_57_S_2_]^2-^ | **5Rha/Fuc + 5GalA/GlcA** | **10** | **2** |
| 15.66 | *1048.7695* | *1048.7676* | 1.81 | [M+C_7_H_15_NH_3_-3H]^2-^ | [C_73_H_119_NO_62_S_3_]^2-^ | **7Rha/Fuc + 4GalA/GlcA** | **11** | **3** |
| 15.66 | *1121.7985* | *1121.7965* | 1.78 | [M+C_7_H_15_NH_3_-3H]^2-^ | [C_79_H_129_NO_66_S_3_]^2-^ | **8Rha/Fuc + 4GalA/GlcA** | **12** | **3** |
| 15.73 | *893.1812* | *893.1793* | 2.13 | [M-2H]^2-^ | [C_60_H_90_O_57_S_2_]^2-^ | **5Rha/Fuc + 5GalA/GlcA** | **10** | **2** |
| 15.73 | *1048.7695* | *1048.7676* | 1.81 | [M+C_7_H_15_NH_3_-3H]^2-^ | [C_73_H_119_NO_62_S_3_]^2-^ | **7Rha/Fuc + 4GalA/GlcA** | **11** | **3** |
| 15.73 | *1127.2549* | *1127.2532* | 1.51 | [M-2H]^2-^ | [C_78_H_118_O_71_S_2_]^2-^ | **7Rha/Fuc + 6GalA/GlcA** | **12** | **3** |
| 15.83 | *820.1507* | *820.1503* | 0.49 | [M-2H]^2-^ | [C_54_H_80_O_53_S_2_]^2-^ | **4Rha/Fuc + 5GalA/GlcA** | **9** | **1** |
| 15.83 | *863.2054* | *863.2051* | 0.35 | [M-2H]^2-^ | [C_60_H_94_O_53_S_2_]^2-^ | **6Rha/Fuc + 3GalA/GlcA** | **10** | **2** |
| 15.83 | *893.1795* | *893.1793* | 0.22 | [M-2H]^2-^ | [C_60_H_90_O_57_S_2_]^2-^ | **5Rha/Fuc + 5GalA/GlcA** | **10** | **1** |
| 15.83 | *966.2065* | *966.2082* | 1.76 | [M-2H]^2-^ | [C_66_H_100_O_61_S_2_]^2-^ | **6Rha/Fuc + 5GalA/GlcA** | **11** | **1** |
| 15.83 | *1054.2242* | *1054.2243* | 0.09 | [M-2H]^2-^ | [C_72_H_108_O_67_S_2_]^2-^ | **6Rha/Fuc + 6GalA/GlcA** | **12** | **1** |
| 15.95 | *556.1041* | *556.1022* | 3.42 | [M-2H]^2-^ | [C_36_H_56_O_35_S_2_]^2-^ | **4Rha/Fuc + 2GalA/GlcA** | **6** | **2** |
| 15.95 | *790.1784* | *790.1761* | 2.91 | [M-2H]^2-^ | [C_54_H_84_O_49_S_2_]^2-^ | **6Rha/Fuc + 3GalA/GlcA** | **9** | **2** |
| 15.95 | *985.2719* | *985.2719* | 0 | [M+C_7_H_15_NH_3_-3H]^2-^ | [C_68_H_118_N_2_O_55_S_4_]^2-^ | **6Rha/Fuc + 3GalA/GlcA** | **9** | **4** |
| 15.95 | *1146.3173* | *1146.314* | 2.88 | [M+C_7_H_15_NH_3_-3H]^2-^ | [C_80_H_136_N_2_O_65_S_4_]^2-^ | **7Rha/Fuc + 4GalA/GlcA** | **11** | **4** |
| 15.95 | *1219.3455* | *1219.343* | 2.05 | [M+C_7_H_15_NH_3_-3H]^2-^ | [C_86_H_146_N_2_O_69_S_4_]^2-^ | **8Rha/Fuc + 4GalA/GlcA** | **12** | **4** |
| 15.95 | *1224.8026* | *1224.7997* | 2.37 | [M+C_7_H_15_NH_3_-3H]^2-^ | [C_85_H_135_NO_74_S_3_]^2-^ | **7Rha/Fuc + 6GalA/GlcA** | **13** | **2** |
| 15.95 | *1297.8292* | *1297.8286* | 0.46 | [M+C_7_H_15_NH_3_-3H]^2-^ | [C_91_H_145_NO_78_S_3_]^2-^ | **8Rha/Fuc + 6GalA/GlcA** | **14** | **3** |
| 15.95 | *1307.356* | *1307.359* | 2.29 | [M+C_7_H_15_NH_3_-3H]^2-^ | [C_92_H_154_N_2_O_75_S_4_]^2-^ | **8Rha/Fuc + 5GalA/GlcA** | **13** | **4** |
| 15.95 | *1380.3879* | *1380.388* | 0.07 | [M+C_7_H_15_NH_3_-3H]^2-^ | [C_98_H_164_N_2_O_79_S_4_]^2-^ | **9Rha/Fuc + 5GalA/GlcA** | **14** | **4** |
| 15.95 | *1453.4143* | *1453.417* | 1.86 | [M+C_7_H_15_NH_3_-3H]^2-^ | [C_104_H_174_N_2_O_83_S_4_]^2-^ | **10Rha/Fuc + 5GalA/GlcA** | **15** | **4** |
| 15.95 | *1458.8691* | *1458.8736* | 3.08 | [M+C_7_H_15_NH_3_-3H]^2-^ | [C_103_H_163_NO_88_S_3_]^2-^ | **9Rha/Fuc + 7GalA/GlcA** | **16** | **3** |
| 16.02 | *551.6015* | *551.5995* | 3.63 | [M-4H]^4-^ | [C_72_H_110_O_69_S_4_]^4-^ | **8Rha/Fuc + 4GalA/GlcA** | **12** | **4** |
| 16.02 | *624.1115* | *624.1098* | 2.72 | [M-2H]^2-^ | [C_42_H_56_O_43_]^2-^ | **7GalA/GlcA** | **7** | **0** |
| 16.02 | *668.639* | *668.6365* | 3.74 | [M-4H]^4-^ | [C_90_H_138_O_83_S_4_]^4-^ | **10Rha/Fuc + 5GalA/GlcA** | **15** | **4** |
| 16.02 | *709.1519* | *709.1497* | 3.1 | [M-3H]^3-^ | [C_72_H_111_O_66_S_3_]^3-^ | **8Rha/Fuc + 4GalA/GlcA** | **12** | **3** |
| 16.02 | *785.1563* | *785.1548* | 1.91 | [M-2H]^2-^ | [C_54_H_74_O_53_]^2-^ | **Rha/Fuc + 8GalA/GlcA** | **9** | **0** |
| 16.02 | *885.1841* | *885.1818* | 2.6 | [M-3H]^3-^ | [C_90_H_135_O_84_S_3_]^3-^ | **8Rha/Fuc + 4GalA/GlcA** | **13** | **3** |
| 16.02 | *995.2641* | *995.261* | 3.11 | [M+C_7_H_15_NH_3_-4H]^3-^ | [C_104_H_173_N_2_O_86_S_5_]^3-^ | **10Rha/Fuc + 5GalA/GlcA** | **15** | **5** |
| 16.02 | *1151.3129* | *1151.3103* | 2.26 | [M+C_7_H_15_NH_3_-4H]^3-^ | [C_122_H_201_N_2_O_100_S_5_]^3-^ | **12Rha/Fuc + 6GalA/GlcA** | **18** | **5** |
| 16.02 | *1316.892* | *1316.8895* | 1.9 | [M+C_7_H_15_NH_3_-3H]^2-^ | [C_93_H_163_N_3_O_72_S_5_]^2-^ | **8Rha/Fuc + 4GalA/GlcA** | **12** | **5** |
| 16.02 | *1550.9541* | *1550.9634* | 6 | [M+C_7_H_15_NH_3_-3H]^2-^ | [C_111_H_191_N_3_O_88_S_5_]^2-^ | **10Rha/Fuc + 5GalA/GlcA** | **15** | **5** |
| 16.12 | *712.1271* | *712.1258* | 1.83 | [M-2H]^2-^ | [C_48_H_62_O_49_]^2-^ | **8GalA/GlcA** | **8** | **0** |
| 16.12 | *873.171* | *873.1708* | 0.23 | [M-2H]^2-^ | [C_60_H_82_O_59_]^2-^ | **Rha/Fuc + 9GalA/GlcA** | **10** | **0** |
| 16.12 | *1082.8177* | *1082.8155* | 2.03 | [M+C_7_H_15_NH_3_-3H]^2-^ | [C_75_H_135_N_3_O_58_S_5_]^2-^ | **6Rha/Fuc + 3GalA/GlcA** | **9** | **5** |
| 16.12 | *1216.3446* | *1216.3412* | 2.8 | [M+C_7_H_15_NH_3_-4H]^3-^ | [C_129_H_218_N_3_O_103_S_6_]^3-^ | **12Rha/Fuc + 6GalA/GlcA** | **18** | **6** |
| 16.12 | *1316.8921* | *1316.8895* | 1.97 | [M+C_7_H_15_NH_3_-3H]^2-^ | [C_75_H_135_N_3_O_58_S_5_]^2-^ | **8Rha/Fuc + 4GalA/GlcA** | **12** | **5** |
| 16.12 | *1372.3914* | *1372.3905* | 0.66 | [M+C_7_H_15_NH_3_-4H]^3-^ | [C_147_H_246_N_3_O_117_S_6_]^3-^ | **14Rha/Fuc + 7GalA/GlcA** | **21** | **6** |
| 16.18 | *800.1444* | *800.1419* | 3.12 | [M-2H]^2-^ | [C_54_H_72_O_55_]^2-^ | **9GalA/GlcA** | **9** | **0** |
| 16.18 | *961.1846* | *961.1869* | 2.39 | [M-2H]^2-^ | [C_66_H_90_O_65_]^2-^ | **Rha/Fuc + 10GalA/GlcA** | **11** | **0** |
| 16.18 | *1122.2288* | *1122.2319* | 2.76 | [M-2H]^2-^ | [C_78_H_108_O_75_]^2-^ | **2Rha/Fuc + 11GalA/GlcA** | **13** | **0** |
| 16.25 | *888.1598* | *888.1579* | 2.14 | [M-2H]^2-^ | [C_60_H_80_O_61_]^2-^ | **10GalA/GlcA** | **10** | **0** |
| 16.25 | *1049.1985* | *1049.2029* | 4.19 | [M-2H]^2-^ | [C_72_H_98_O_71_]^2-^ | **Rha/Fuc + 11GalA/GlcA** | **12** | **0** |
| 16.3 | *976.1754* | *976.1739* | 1.54 | [M-2H]^2-^ | [C_66_H_88_O_67_]^2-^ | **11GalA/GlcA** | **11** | **0** |
| 16.3 | *1049.1985* | *1049.2029* | 4.19 | [M-2H]^2-^ | [C_72_H_98_O_71_]^2-^ | **Rha/Fuc + 11GalA/GlcA** | **12** | **0** |
| 16.3 | *1137.2118* | *1137.2189* | 6.24 | [M-2H]^2-^ | [C_78_H_106_O_77_]^2-^ | **Rha/Fuc + 12GalA/GlcA** | **13** | **0** |

Rha : Rhamnose ; Fuc : Fucose ; GalA : galacturonic acid ; GlcA : Glucuronic acid ; Gal : Galactose ; Glu : glucose

* Rhamnose/fucose, galacturonic acid/glucuronic acid and galactose/glucose being isobaric, they cannot be distinguished by MS

Table S2 : Resolution of all the fragments of the 7 characteristic ions

| **Precursor ion (m/z)** | **Collision energy (eV)** | **Fragment** | **Fragment ion (m/z)** | | | |
| --- | --- | --- | --- | --- | --- | --- |
|  |  |  | ***Experimental value*** | ***Predicted value*** | **Resolution (ppm)** |  |
| **679.1935** | **40** | **[M-H]^-^** | *339.093* | *339.0933* | 0.88 |  |
|  |  | **[M-H_2_O]^-^** | *321.082* | *321.0827* | 2.18 |  |
|  |  | **B** | *145.0496* | *145.0506* | 6.89 |  |
|  |  | **Y** | *193.035* | *193.0354* | 2.07 |  |
|  |  | **Z** | *175.0241* | *175.0248* | 4.00 |  |
|  |  | **^0,2^X_1_** | *235.0469* | *235.0452* | 7.23 |  |
|  |  | **^0,3^X_1_** | *265.0403* | *265.0565* | 61.12 |  |
|  |  | **^1,3^X_1_ or ^2,4^X_1_** | *279.0698* | *279.0722* | 8.60 |  |

| **Precursor ion (m/z)** | **Collision energy (eV)** | **Fragment** | **Fragment ion (m/z)** | | |
| --- | --- | --- | --- | --- | --- |
|  |  |  | ***Experimental value*** | ***Predicted value*** | **Resolution (ppm)** |
| **535.1371** | **40** | **B_1_ or Z_1_** | *225.0084* | *225.0074* | 4.44 |
|  |  | **B_1_ or Z_1_ (- SO_3_)** | *145.0504* | *145.0506* | 1.38 |
|  |  | **Y_1_** | *243.019* | *243.018* | 4.11 |
|  |  | **Y_2_** | *389.0774* | *389.0759* | 3.86 |
|  |  | **Z_2_** | *371.0665* | *371.0654* | 2.96 |

| **Precursor ion (m/z)** | **Collision energy (eV)** | **Fragment** | **Fragment ion (m/z)** | | |
| --- | --- | --- | --- | --- | --- |
|  |  |  | ***Experimental value*** | ***Predicted value*** | **Resolution (ppm)** |
| **973.3090** | **60** | **B_1_ or Z_1_ (- SO_3_)** | *145.0504* | *145.0506* | 1.38 |
|  |  | **B_1_ or Z_1_** | *225.0074* | *225.0074* | 0.00 |
|  |  | **Y_1_ or C_1_** | *243.0181* | *243.018* | 0.41 |
|  |  | **Y_2_** | *389.0768* | *389.0759* | 2.31 |
|  |  | **Y_3_** | *535.134* | *535.1338* | 0.37 |
|  |  | **Y_4_** | *681.1925* | *681.1917* | 1.17 |
|  |  | **Y_5_** | *827.2515* | *827.2496* | 2.30 |
|  |  | **Z_5_** | *809.2377* | *809.2391* | 1.73 |

| **Precursor ion (m/z)** | **Collision energy (eV)** | **Fragment** | **Fragment ion (m/z)** | | |  |
| --- | --- | --- | --- | --- | --- | --- |
|  |  |  | ***Experimental value*** | ***Predicted value*** | **Resolution (ppm)** |  |
| **1149.3435** | **60** | **[M-SO_3_]^-^** | *1069.3845* | *1069.3828* | 1.59 |  |
|  |  | **B_1_** | *175.0231* | *175.0248* | 9.71 |  |
|  |  | **B_2_** | *321.081* | *321.0827* | 5.29 |  |
|  |  | **B_5_** | *759.2562* | *759.2564* | 0.26 |  |
|  |  | **B_6_** | *905.3168* | *905.3144* | 2.65 |  |
|  |  | **C_1_** | *193.0345* | *193.0354* | 4.66 |  |
|  |  | **C_2_** | *339.0923* | *339.0933* | 2.95 |  |
|  |  | **C_3_** | *485.1519* | *485.1512* | 1.44 |  |
|  |  | **C_6_** | *923.3267* | *923.3249* | 1.95 |  |
|  |  | **Y_1_** | *243.018* | *243.018* | 0.00 |  |
|  |  | **Y_1_ (- SO_3_)** | *163.06* | *163.0612* | 7.36 |  |
|  |  | **Y_2_** | *389.0776* | *389.0759* | 4.37 |  |
|  |  | **Y_2_ (- SO_3_)** | *309.1199* | *309.1191* | 2.59 |  |
|  |  | **Y_6_** | *827.2522* | *827.2496* | 3.14 |  |
|  |  | **Z_1_** | *225.0063* | *225.0074* | 4.89 |  |
|  |  | **Z_1_ (- SO_3_)** | *145.0489* | *145.0506* | 11.72 |  |
|  |  | **Z_2_** | *371.0641* | *371.0654* | 3.50 |  |
|  |  | **Z_2_ (- SO_3_)** | *291.1088* | *291.1085* | 1.03 |  |
|  |  | **Z_3_** | *517.122* | *517.1233* | 2.51 |  |
|  |  | **Z_3_ (- SO_3_)** | *437.1627* | *437.1664* | 8.46 |  |
|  |  | **Z_4_** | *663.1808* | *663.1812* | 0.60 |  |
|  |  | **Z_5_** | *809.2409* | *809.2391* | 2.22 |  |
|  |  | **Z_6_** | *955.2956* | *955.297* | 1.47 |  |
|  |  | **^0,2^X_6_** | *1015.3167* | *1015.3181* | 1.38 |  |
|  |  | **^0,2^A_6_ or ^1,3^X_6_** | *133.0139* | *133.0142* | 2.26 |  |
|  |  | **^0,4^A_6_** | *72.9917* | *72.9931* | 19.18 |  |
|  |  | **^0,4^X_6_** | *1075.3033* | *1075.3392* | 33.38 |  |
| **Precursor ion (m/z)** | **Collision energy (eV)** | **Fragment** | **Fragment ion (m/z)** | | | |
|  |  |  | ***Experimental value*** | ***Predicted value*** | **Resolution (ppm)** | |
| **1085.2172** | **40 or 60** | **[M-SO_3_]^-^** | *175.0231* | *175.0248* | 9.71 | |
|  |  | **B_1_** | *197.0082* | *197.0068* | 7.11 | |
|  |  | **B_1_ (- Na)** | *175.0247* | *175.0248* | 0.57 | |
|  |  | **B_2_** | *321.0839* | *321.0827* | 3.74 | |
|  |  | **B_3_** | *519.0936* | *519.0968* | 6.16 | |
|  |  | **B_4_ or Z_4_ (- SO_3_)** | *665.1605* | *665.1547* | 8.72 | |
|  |  | **B_5_** | *841.1904* | *841.1867* | 4.40 | |
|  |  | **C_1_** | *215.014* | *215.0173* | 15.35 | |
|  |  | **C_2_ or Y_2_ (- SO_3_)** | *361.0744* | *361.0752* | 2.22 | |
|  |  | **C_3_** | *537.1041* | *537.1073* | 5.96 | |
|  |  | **C_4_ or Y_4_ (- SO_3_)** | *683.1624* | *683.1652* | 4.10 | |
|  |  | **C_5_** | *859.1928* | *859.1973* | 5.24 | |
|  |  | **Y_1_** | *243.0188* | *243.018* | 3.30 | |
|  |  | **Y_2_** | *441.0328* | *441.032* | 1.82 | |
|  |  | **Y_3_** | *587.09* | *587.09* | 0.00 | |
|  |  | **Y_4_** | *763.1222* | *763.122* | 0.26 | |
|  |  | **Z_1_** | *225.0029* | *225.0074* | 20.00 | |
|  |  | **Z_1_ (- SO_3_)** | *145.0487* | *145.0506* | 13.10 | |
|  |  | **Z_2_** | *423.0185* | *423.0215* | 7.10 | |
|  |  | **Z_2_ (- SO_3_)** | *343.0648* | *343.0647* | 0.29 | |
|  |  | **Z_3_** | *569.0784* | *569.0794* | 1.76 | |
|  |  | **Z_4_** | *745.1122* | *745.1115* | 0.94 | |
|  |  | **^1,3^A_3_** | *403.0874* | *403.0858* | 3.97 | |
|  |  | **^1,3^X_3_** | *623.1444* | *623.1441* | 0.48 | |

| **Precursor ion (m/z)** | **Collision energy (eV)** | **Fragment** | **Fragment ion (m/z)** | | |
| --- | --- | --- | --- | --- | --- |
|  |  |  | ***Experimental value*** | ***Predicted value*** | **Resolution (ppm)** |
| **773.1838** | **40** | **B_1_ or Z_1_** | *175.0239* | *175.0248* | 5.14 |
|  |  | **B_2_ or Z_2_** | *321.0805* | *321.0827* | 6.85 |
|  |  | **B_3_** | *483.136* | *483.1355* | 1.03 |
|  |  | **B_4_** | *659.1591* | *659.1676* | 12.80 |
|  | **Ramp 20-40** | **B_5_** | *805.2282* | *805.2255* | 3.35 |
|  |  | **B_6_ (- SO_3_)** | *951.2896* | *951.2834* | 6.52 |
|  |  | **B_7_ or Z_7_ (- SO_3_)** | *1127.3296* | *1127.3155* | 12.51 |
|  |  | **B_8_ (- SO_3_)** | *1273.4075* | *1273.3734* | 26.78 |
|  |  | **B_8_ or Z_8_** | *1353.3413* | *1353.3303* | 8.13 |
|  | **40** | **C_1_ or Y_1_** | *193.0343* | *193.0354* | 5.70 |
|  |  | **C_2_ or Y_2_** | *339.0925* | *339.0933* | 2.36 |
|  |  | **C_3_** | *501.1396* | *501.1461* | 12.97 |
|  | **Ramp 20-40** | **C_5_** | *823.2365* | *823.2361* | 0.49 |
|  |  | **C_6_** | *1049.2524* | *1049.2508* | 1.52 |
|  |  | **C_6_ (- SO_3_)** | *969.2903* | *969.294* | 3.82 |
|  |  | **C_7_ or Y_7_** | *1225.2858* | *1225.2829* | 2.367 |
|  |  | **C_7_ or Y_7_ (- SO_3_)** | *1145.3337* | *1145.3261* | 6.64 |
|  |  | **C_8_ or Y_8_** | *1371.3553* | *1371.3408* | 10.57 |
|  |  | **C_8_ or Y_8_ (- SO_3_)** | *1291.4006* | *1291.384* | 12.85 |
|  | **40** | **Y_3_** | *515.1243* | *515.1254* | 2.14 |
|  |  | **Y_4_** | *661.1927* | *661.1833* | 14.22 |
|  |  | **Y_4_** | *741.1385* | *741.1401* | 2.12 |
|  | **Ramp 20-40** | **Y_5_** | *887.2088* | *887.198* | 12.17 |
|  | **40** | **Z_3_** | *497.1159* | *497.1148* | 2.21 |
|  |  | **Z_4_** | *643.1721* | *643.1727* | 0.93 |

| **Precursor ion (m/z)** | **Collision energy (eV)** | **Fragment** | **Fragment ion (m/z)** | | |
| --- | --- | --- | --- | --- | --- |
|  |  |  | ***Experimental value*** | ***Predicted value*** | **Resolution (ppm)** |
| **1137.2118** | **40** | **B_1_ or Z_1_** | *175.0243* | *175.0248* | 2.86 |
|  |  | **B_2_ or Z_2_** | *351.0554* | *351.0569* | 4.27 |
|  |  | **B_3_** | *497.114* | *497.1148* | 1.61 |
|  |  | **B_4_** | *673.142* | *673.1469* | 7.28 |
|  |  | **B_5_** | *849.1735* | *849.179* | 6.48 |
|  |  | **B_6_** | *1025.2037* | *1025.2111* | 7.22 |
|  |  | **B_7_** | *1201.2429* | *1201.2432* | 0.25 |
|  |  | **B_8_** | *1377.2688* | *1377.2752* | 4.65 |
|  |  | **B_9_** | *1553.3167* | *1553.3073* | 6.052 |
|  |  | **B_10_** | *1729.3452* | *1729.3394* | 3.35 |
|  |  | **B_11_ or Z_11_** | *1905.3761* | *1905.3715* | 2.41 |
|  |  | **C_1_ or Y_1_** | *193.0345* | *193.0354* | 4.66 |
|  |  | **C_2_ or Y_2_** | *369.0664* | *369.0675* | 2.98 |
|  |  | **C_3_** | *515.1239* | *515.1254* | 2.92 |
|  |  | **C_4_** | *691.1559* | *691.1575* | 2.31 |
|  |  | **C_5_** | *867.188* | *867.1895* | 1.73 |
|  |  | **C_6_** | *1043.2223* | *1043.2216* | 0.67 |
|  |  | **C_7_** | *1219.2544* | *1219.2537* | 0.57 |
|  |  | **C_8_** | *1395.2732* | *1395.2858* | 9.03 |
|  |  | **C_9_** | *1571.3251* | *1571.3179* | 4.58 |
|  |  | **C_10_** | *1747.3344* | *1747.35* | 8.93 |
|  |  | **C_11_ or Y_11_** | *1923.4143* | *1923.3821* | 16.74 |
|  |  | **Y_3_** | *545.0975* | *545.0996* | 3.85 |
|  |  | **Y_4_** | *721.1292* | *721.1316* | 3.33 |
|  |  | **Y_5_** | *897.1581* | *897.1637* | 6.24 |
|  |  | **Y_6_** | *1073.1936* | *1073.1958* | 2.05 |
|  |  | **Y_7_** | *1249.2323* | *1249.2279* | 3.52 |
|  |  | **Y_8_** | *1425.2715* | *1425.26* | 8.07 |
|  |  | **Y_9_** | *1601.2391* | *1601.2921* | 33.10 |
|  |  | **Y_10_** | *1777.3035* | *1777.3242* | 11.65 |
|  |  | **Z_3_** | *527.0879* | *527.089* | 2.09 |
|  |  | **Z_4_** | *703.1177* | *703.1211* | 4.84 |
|  |  | **Z_5_** | *879.1475* | *879.1532* | 6.48 |
|  |  | **Z_6_** | *1055.1818* | *1055.1853* | 3.32 |
|  |  | **Z_7_** | *1231.2151* | *1231.2173* | 1.79 |
|  |  | **Z_8_** | *1407.2494* | *1407.2406* | 6.25 |
|  |  | **Z_9_** | *1583.309* | *1583.2815* | 17.37 |
|  |  | **Z_10_** | *1759.3247* | *1759.3136* | 6.31 |

Table S3 : Values of R² obtained for the two models (Herschel-Bulkley and power-low)

|  | **ESP-3707** | | **ESP-3688** | |
| --- | --- | --- | --- | --- |
| **Concentration** | R^2^ (Herschel-Bulkley) | R^2^ (power-law) | R^2^ (Herschel-Bulkley) | R^2^ (power-law) |
| 0.5 g L^-1^ | 0.996 | 0.981 | 0.995 | 0.982 |
| 1 g L^-1^ | 0.994 | 0.978 | 0.996 | 0.980 |
| 5 g L^-1^ | 0.995 | 0.963 | 0.993 | 0.984 |
| 10 g L^-1^ | 0.992 | 0.951 | 0.991 | 0.979 |

Figure S1 : MS/MS spectra of characteristics ions for each fraction

**679.1935**

**535.1371**

**973.309**

**1149.3435**

**1085.2172**

**773.1838**

**1137.2118**
